# TCF7L2 regulates postmitotic differentiation programmes and excitability patterns in the thalamus

**DOI:** 10.1242/dev.190181

**Published:** 2020-08-25

**Authors:** Marcin Andrzej Lipiec, Joanna Bem, Kamil Koziński, Chaitali Chakraborty, Joanna Urban-Ciećko, Tomasz Zajkowski, Michał Dąbrowski, Łukasz Mateusz Szewczyk, Angel Toval, José Luis Ferran, Andrzej Nagalski, Marta Barbara Wiśniewska

**Affiliations:** 1 Centre of New Technologies, University of Warsaw, Banacha 2, 02-097 Warsaw, Poland; 2 Faculty of Biology, University of Warsaw, Miecznikowa 1, 02-096 Warsaw, Poland; 3 Nencki Institute of Experimental Biology, Pasteur 3, 02-093 Warsaw, Poland; 4 Department of Human Anatomy and Psychobiology, School of Medicine, University of Murcia and IMIB-Arrixaca Institute, Campus de la Salud, 30120 El Palmar, Murcia, Spain

**Keywords:** TCF7L2, Thalamus, Brain development, Neuronal identity, Terminal selector, Transcription factor

## Abstract

Neuronal phenotypes are controlled by terminal selector transcription factors in invertebrates, but only a few examples of such regulators have been provided in vertebrates. We hypothesised that TCF7L2 regulates different stages of postmitotic differentiation in the thalamus, and functions as a thalamic terminal selector. To investigate this hypothesis, we used complete and conditional knockouts of *Tcf7l2* in mice. The connectivity and clustering of neurons were disrupted in the thalamo-habenular region in *Tcf7l2^−/−^* embryos. The expression of subregional thalamic and habenular transcription factors was lost and region-specific cell migration and axon guidance genes were downregulated. In mice with a postnatal *Tcf7l2* knockout, the induction of genes that confer thalamic terminal electrophysiological features was impaired. Many of these genes proved to be direct targets of TCF7L2. The role of TCF7L2 in terminal selection was functionally confirmed by impaired firing modes in thalamic neurons in the mutant mice. These data corroborate the existence of master regulators in the vertebrate brain that control stage-specific genetic programmes and regional subroutines, maintain regional transcriptional network during embryonic development, and induce terminal selection postnatally.

## Abstract (precis)

**Highlighted Article:** This study describes a role of TCF7L2 in neuronal differentiation of thalamic glutamatergic neurons at two developmental stages, highlighting its involvement in the postnatal establishment of crucial thalamic electrophysiological features.

## INTRODUCTION

Studies of invertebrates have shown that terminal differentiation gene batteries in individual classes of neurons are induced and maintained by specific transcription factors called terminal selectors, which are expressed throughout life (Hobert and Kratsios, 2019). However, regulatory strategies of postmitotic maturation and terminal selection in vertebrates are unclear. Up until now, only a few terminal selectors have been identified in vertebrates (Cho et al., 2014; Flames and Hobert, 2009; Kadkhodaei et al., 2009; Liu et al., 2010; Lodato et al., 2014; Wyler et al., 2016).

Neurons of the thalamus and habenula are derived from a single progenitor domain (prosomere 2) and are glutamatergic (Watson et al., 2012), except for GABAergic interneurons in the rostral thalamus (Evangelio et al., 2018). The thalamus is a sensory relay centre and part of the cortico-subcortical loops that process sensorimotor information and produce goal-directed behaviours (Sherman, 2017). The habenula controls reward- and aversion-driven behaviours by connecting cortical and subcortical regions with the monoamine system in the brainstem (Benekareddy et al., 2018; Hikosaka, 2010). During postmitotic differentiation, thalamic and habenular neurons segregate into discrete nuclei (Shi et al., 2017; Wong et al., 2018), develop a variety of subregional identities (Guo and Li, 2019; Nakagawa, 2019; Phillips et al., 2019), extend axons toward their targets (Hikosaka et al., 2008; López-Bendito, 2018) and acquire electrophysiological characteristics postnatally (Yuge et al., 2011). The knowledge of the mechanisms that control postmitotic development in this region is important, because its functional dysconnectivity, which possibly originates from the period of postmitotic maturation, is implicated in schizophrenia, autism and other mental disorders (Browne et al., 2018; Steullet, 2019; Whiting et al., 2018; Woodward et al., 2017).

The network of postmitotically induced transcription factors that regulate the maturation of prosomere 2 neurons has only recently begun to be deciphered. *Gbx2* and *Pou4f1* are early postmitotic markers of thalamic and habenular neurons, respectively. GBX2 plays a transient regulatory role in the initial acquisition of thalamic molecular identities and thalamocortical axon guidance, and then its expression is downregulated in the majority of the thalamus (Chatterjee et al., 2012; Chen et al., 2009; Li et al., 2012; Mallika et al., 2015; Miyashita-Lin et al., 1999). In contrast, POU4F1 (also known as BRN3A) is not essential for the growth of habenular axons, but it maintains the expression of the glutamate transporter gene *Vglut1* (also known as *Slc17a7*) and other habenula-abundant genes in adults, though its impact on electrophysiological responses of habenular neurons was not tested (Quina et al., 2009; Serrano-Saiz et al., 2018). Subregional transcription factors RORA and FOXP2 regulate some aspects of postmitotic differentiation in thalamic subregions during embryogenesis (Ebisu et al., 2016; Quina et al., 2009; Vitalis et al., 2017), but their role in terminal differentiation in this region was not investigated*.*

*Tcf7l2*, a risk gene for schizophrenia and autism (Bem et al., 2019) that encodes a member of the LEF1/TCF transcription factor family (Cadigan and Waterman, 2012), is the only shared marker of prosomere 2 neurons (Nagalski et al., 2013, 2016). Its function was tested only during the early postmitotic period in zebrafish (Beretta et al., 2013; Hüsken et al., 2014) and mice (Lee et al., 2017; Tran et al., 2020). In *Tcf7l2^−/−^* mouse embryos, some markers were mis-expressed in the region of prosomere 2, and the formation of axonal tracts was disrupted. TCF7L2 expression is maintained throughout life and the TCF7L2 motif is overrepresented in putative enhancers of adult thalamus-enriched genes (Nagalski et al., 2016; Wisniewska et al., 2012), suggesting that this factor can also play a role of prosomere 2 terminal selector.

The present study used complete and conditional knockout mice to explore the role of *Tcf7l2* in postmitotic anatomical maturation, maintenance of molecular diversification, adoption of neurotransmitter identity and postnatal acquisition of electrophysiological features in the thalamus and habenula. We show that TCF7L2 orchestrates the overall morphological differentiation process in this region by regulating stage-specific gene expression directly or via subregional transcription factors. We also report that TCF7L2 functions as a terminal selector of postnatally induced thalamic electrophysiological characteristics but not glutamatergic (VGLUT2) identity.

## RESULTS

### Generation of mice with the complete and conditional knockouts of *Tcf7l2*

**Fig. 1.**
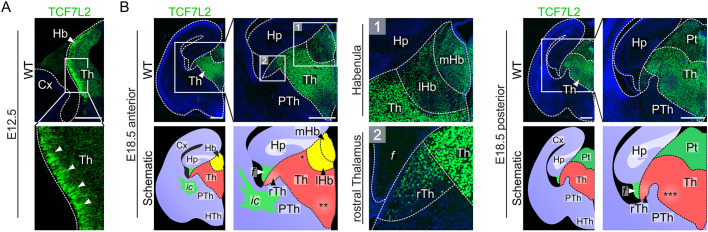
**TCF7L2 protein levels are high in postmitotic neurons in the thalamo-habenular region.** (A) Immunofluorescent staining of TCF7L2 in coronal brain sections from E12.5 embryos. Arrowheads show TCF7L2 in the mantle zone. (B) Immunofluorescent staining of TCF7L2 in coronal brain sections from E18.5 embryos. Asterisks mark nuclei of the caudal thalamus with relatively lower levels of TCF7L2 protein: *, perihabenula; **, nucleus reuniens; ***, ventrobasal complex. Dotted lines demarcate different regions. Magnification of boxed areas are shown as indicated. Cx, cortex; f, fornix; Hb, habenula; Hp, hippocampus; HTh, hypothalamus; ic, internal capsule; lHb, lateral habenula; mHb, medial habenula; Pt, pretectum; PTh, prethalamus; rTh, rostral thalamus; Th, thalamus. Scale bars: 0.25 mm (A); 0.5 mm (B).

*Tcf7l2* expression in prosomere 2 is induced in mice during neurogenesis (Cho and Dressler, 1998) and maintained in the thalamus and medial habenula throughout life (Nagalski et al., 2013). In wild-type (WT) embryos on embryonic day (E)12.5, we observed high levels of TCF7L2 protein in the superficial portion of the thalamus and habenula, which is populated by postmitotic neurons (Fig. 1A). We also observed several TCF7L2-positive cells in the prethalamus. Possibly, these cells migrate from the GABAergic rostral thalamus to take part in the formation of the intergeniculate leaflet and ventral lateral geniculate nuclei that derive from prethalamic and rostral thalamic progenitors (Jeong et al., 2011). At late gestation, TCF7L2 was observed in the entire caudal thalamus (hereinafter referred to as the thalamus; a glutamatergic domain) and medial habenula (Fig. 1B). Relatively lower levels of TCF7L2 were present in the ventrobasal complex (VB), nucleus reuniens, and recently identified perihabenula (Fernandez et al., 2018). Low levels of TCF7L2 were also present in the lateral habenula and the derivatives of the rostral thalamus.

**Fig. 2.**
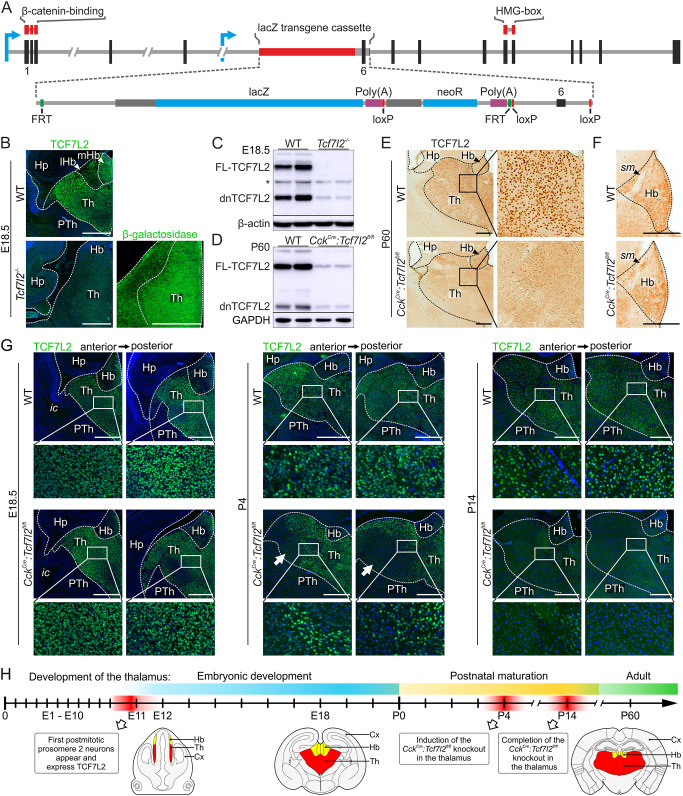
**Generation of *Tcf7l2^−/−^* and *Cck^Cre^:Tcf7l2^fl/fl^* mouse strains.** (A) Schematic showing *Tcf7l2^tm1a^* allele generated by EUCOMM, in which a trap cassette with the *lacZ* and *neoR* elements was inserted upstream of the critical exon 6 of the *Tcf7l2* gene. Exons and introns are represented by vertical black and horizontal grey lines, respectively. Blue arrows indicate transcription start sites. Regions that encode the β-catenin binding domain and HMG-box are marked by red lines above the exons. (B) Immunofluorescent staining of TCF7L2 and β-galactosidase in coronal brain sections from E18.5 WT and *Tcf7l2^−/−^* embryos. (C) Western blot analysis of TCF7L2 in the thalamo-habenular region from E18.5 WT and *Tcf7l2^−/−^* embryos. Higher molecular weight bands represent the full-length protein (FL-TCF7L2), lower weight bands represent the dominant negative isoform of TCF7L2 (dnTCF7L2). Asterisk shows signals from an incompletely stripped staining. (D) Western blot analysis of TCF7L2 in the thalamo-habenular region from P60 WT (*Cck^Cre^:Tcf7l2^+/+^*) and *Cck^Cre^:Tcf7l2^fl/fl^* mice. (E) DAB immunohistochemical staining of TCF7L2 in coronal brain sections from P60 WT and *Cck^Cre^:Tcf7l2^fl/fl^* mice. (F) Magnified view of the thalamus and habenula. (G) Immunofluorescent staining of TCF7L2 in coronal sections from WT and *Cck^Cre^:Tcf7l2^fl/fl^* mice on E18.5, P4 and P14, showing the progression of the *Cck^Cre^*-driven knockout. White arrows show the ventrolateral region of the thalamus in which TCF7L2 is depleted by P4. (H) Simplified time course of the development of the thalamus with representative mouse brain schemes. Dotted lines demarcate different regions. Magnification of boxed areas are shown as indicated. Cx, cortex; Hb, habenula; Hp, hippocampus; ic, internal capsule; lHb, lateral habenula; mHb, medial habenula; PTh, prethalamus; sm, stria medullaris; Th, thalamus. Scale bars: 0.5 mm.

To investigate the role of TCF7L2 as a selector of morphological and electrophysiological characteristics of prosomere 2, we used two knockout models in mice. The complete knockout of *Tcf7l2* was generated by insertion of the *tm1a(KOMP)Wtsi* allele with *lacZ* cassette upstream of the crucial sixth exon of the *Tcf7l2* gene (Fig. 2A). This *Tcf7l2*^tm1a^ allele led to the lack of TCF7L2 protein, confirmed by immunostaining and western blot, and ectopic expression of β-galactosidase from the *lacZ* locus (Fig. 2B,C). *Tcf7l2*^−/−^ mice die after birth. To create a thalamus-specific postnatal knockout of *Tcf7l2*, we first crossed *Tcf7l2^+/^*^tm1a^ mice with a flippase-expressing strain and then with mice that expressed Cre recombinase from the cholecystokinin (*Cck*) gene promoter (Fig. 2D,F and Fig. S1). *Cck* is upregulated postnatally and its expression overlaps with the expression of *Tcf7l2* in the thalamus (Nishimura et al., 2015). The expression from the *Cck^Cre^* locus, visualised in the *Cck^Cre^:tdTomato^fl/+^* reporter line, was high in lateral parts of the thalamus, and lower in thalamic medial and midline parts, including the anterodorsal (AD), paraventricular (PV) and parafascicular (PF) nuclei (Fig. S2). *Cck*-driven knockout of *Tcf7l2* was induced postnatally and completed in the thalamus by postnatal day (P)14 (Fig. 2G,H). In the resulting *Cck^Cre^:Tcf7l2*^fl/fl^ mice, TCF7L2 was absent in most thalamic nuclei in adults, except for the PV and PF (Fig. S3). In the AD and midline nuclei, *Tcf7l2* was partially knocked out.

### Normal neurogenesis but disrupted anatomy and connectivity of prosomere 2

**Fig. 3.**
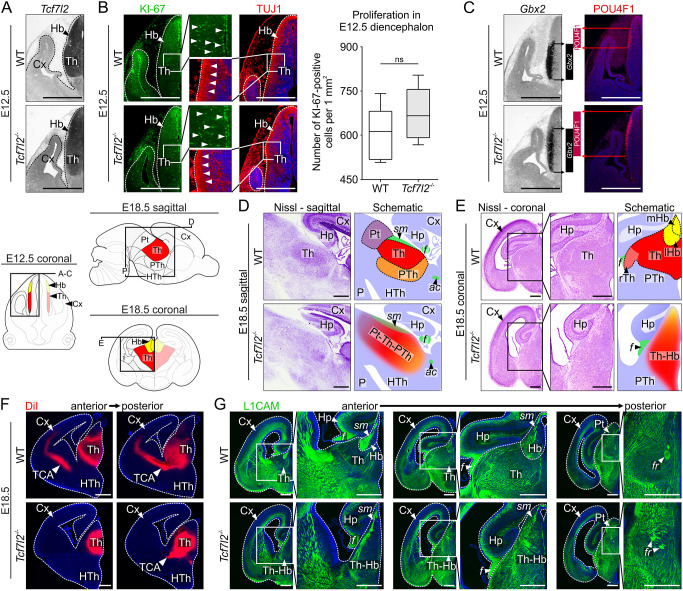
**TCF7L2 controls the establishment of proper anatomy and axonal connections of the thalamo-habenular region at late gestation but does not affect proliferation and neurogenesis.** (A) *In situ* hybridisation with a *Tcf7l2* probe in E12.5 coronal brain sections (truncated *Tcf7l2* mRNA is expressed in *Tcf7l2^−/−^* embryos). Schematic below (E12.5) shows an area sectioned. (B) Immunofluorescent staining of the proliferation marker KI-67 and neural marker TUJ1 in E12.5 coronal brain sections. Right panel shows quantification of proliferating cells in prosomere 2 [WT *n*=3 (four sections each); *Tcf7l2^−/−^ n*=3 (four sections each); two-tailed unpaired Student's *t*-test; *P*-value=0.0615]. (C) *In situ* hybridisation with a *Gbx2* probe and immunofluorescent staining of POU4F1 in consecutive coronal brain sections from the same embryo on E12.5. Spreading of the *Gbx2*-positive and POU4F1-positive regions into each other's territory in *Tcf7l2^−/−^* embryos is emphasised by bars. (D,E) Nissl staining in E18.5 sagittal (D) and coronal (E) brain sections. Schematic on left (E18.5) shows areas sectioned. (F) DiI tracing of thalamocortical tracts in E18.5 brains. (G) Immunofluorescent staining of the axonal marker L1CAM in the thalamo-habenular region on E18.5. Dotted lines demarcate different regions. Magnification of boxed areas are shown as indicated. ac, anterior commissure; Cx, cortex; f, fornix; fr, fasciculus retroflexus; Hb, habenula; Hp, hippocampus; HTh, hypothalamus; ic, internal capsule; lHb, lateral habenula; mHb, medial habenula; P, pons; Pt, pretectum; PTh, prethalamus; rTh, rostral thalamus; sm, stria medullaris; TCA, thalamocortical axons; Th, thalamus; Th-Hb, thalamo-habenular region. Scale bars: 0.25 mm (A-C); 0.5 mm (D-G).

To confirm that proliferation and neurogenesis occurred normally within prosomere 2 in *Tcf7l2*^−/−^ mice, we stained E12.5 brain sections with antibodies specific for the KI-67 (MKI67) antigen and TUJ1 (TUBB3), markers of proliferating progenitors and young postmitotic neurons, respectively. Prosomere 2 was identified with a *Tcf7l2* probe both in WT and knockout (KO) embryos, taking advantage of preserved expression from the targeted *Tcf7l2* locus (Fig. 3A). The complete knockout of *Tcf7l2* did not cause any apparent defects in proliferation or neurogenesis in prosomere 2 on E12.5 (Fig. 3B), consistent with previous results in another *Tcf7l2* knockout strain (Lee et al., 2017).

To investigate whether TCF7L2 is required for the initial acquisition of postmitotic molecular identities in the thalamus and habenula, we examined the expression of the *Gbx2* gene and POU4F1 protein (the earliest markers of postmitotic neurons in the thalamus and habenula, respectively) during neurogenesis. Both *Gbx2* mRNA and POU4F1 were highly expressed in the prosomere 2 area in WT and *Tcf7l2^−/−^* embryos on E12.5, indicating that their expression is not induced by TCF7L2 (Fig. 3C). The number of POU4F1-positive cells visibly increased, suggesting that prosomere 2 cells more readily adopted habenular fate in *Tcf7l2*^−/−^ embryos at this stage. *Gbx2*- and POU4F1-positive areas expanded extensively into each other's territory, implying a defect in thalamo-habenular boundary formation.

To further investigate whether TCF7L2 regulates structural maturation of prosomere 2, we focused on late gestation (E18.5), when nucleogenesis is already concluded and axonal connections are well developed in this region. The anatomy of the area was analysed using Nissl staining (Fig. 3D,E and Fig. S4). The boundaries between prosomere 2 and neighbouring structures, i.e. the prethalamus (rostral boundary) and pretectum (caudal boundary) were not morphologically detected in sagittal sections from *Tcf7l2^−/−^* embryos. On coronal sections, the habenula was fused with the thalamus, and nuclear groups within prosomere 2 were not well demarcated. The whole region was reduced in the radial dimension and elongated dorsoventrally, resulting in an oval-like shape. Then, we analysed circuit formation in the thalamo-habenular region by tracing thalamocortical axons with DiI and immunostaining brain sections with an antibody specific for L1 cell adhesion molecule (L1CAM) that marks growing axons. Consistent with a previous report (Lee et al., 2017), thalamocortical axons were not detected in KO brains (Fig. 3F). The bundles of stria medullaris, which include afferent fibres from the basal forebrain and lateral hypothalamus to the habenula, and were previously reported to be normal in *Tcf7l2^−/−^* embryos on E16.5 (Lee et al., 2017), were disorganised and less compact on E18.5 (Fig. 3G). The major habenular efferent tract, i.e. the fasciculus retroflexus, was split into thinner fascicles. The L1CAM staining also showed major disruption in the general topography of axonal connections in the whole thalamic area. This demonstrated that TCF7L2 is crucially involved in the development of habenular and thalamic anatomy and connectivity.

### Impaired cell sorting in the diencephalon

**Fig. 4.**
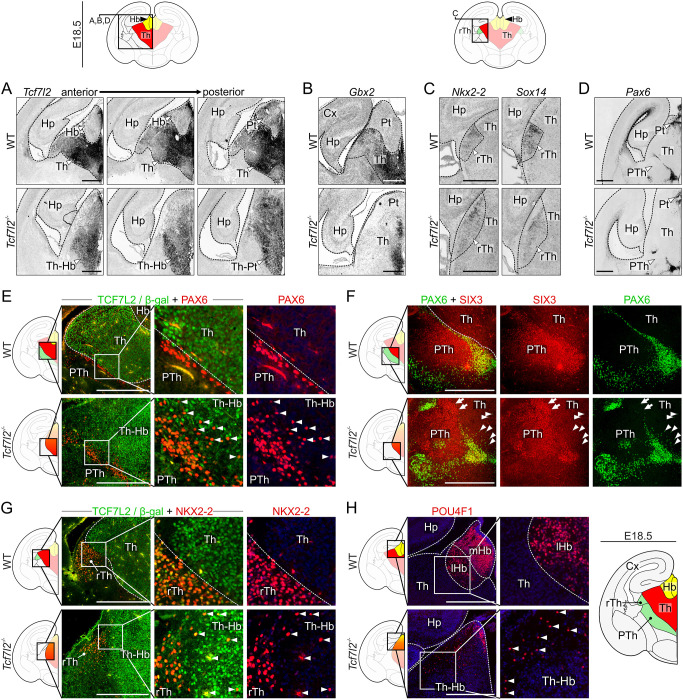
**TCF7L2 controls the establishment of anatomical borders in the thalamus and habenula.** (A) *In situ* hybridisation with a *Tcf7l2* probe in consecutive E18.5 coronal brain sections. (B-D) *In situ* hybridisation with *Gbx2* (B), *Nkx2-2* and *Sox14* (C) and *Pax6* (D) probes in E18.5 coronal brain sections. Schematic above indicates areas shown in micrographs below. (E) Immunofluorescent co-staining of PAX6 and TCF7L2 (WT embryos) or β-galactosidase (*Tcf7l2^−/−^* embryos) in E18.5 coronal brain sections. White arrowheads show PAX6-positive cells invading the thalamus in *Tcf7l2^−/−^* embryos. (F) Immunofluorescent staining of PAX6 and SIX3 in E18.5 coronal brain sections. White arrows show SIX3-positive cells and white arrowheads show PAX6-positive cells which intermingle into the thalamic region. (G) Immunofluorescent co-staining of NKX2-2 and TCF7L2 (WT mice) or β-galactosidase (*Tcf7l2^−/−^* mice) in E18.5 coronal brain sections. White arrowheads show NKX2-2-positive cells from the rostral thalamus invading the thalamus in *Tcf7l2^−/−^* embryos. (H) Immunofluorescent staining of POU4F1 in coronal brain sections. White arrowheads show POU4F1-positive cells spreading into the thalamic area. Dotted lines demarcate different regions. Magnification of boxed areas are shown as indicated. Cx, cortex; Hb, habenula; lHb, lateral habenula; mHb, medial habenula; Hp, hippocampus; Pt, pretectum; PTh, prethalamus; rTh, rostral thalamus; Th, thalamus; Th-Hb, thalamo-habenular region. Scale bars: 0.5 mm.

Because anatomical boundaries of prosomere 2 were blurred in *Tcf7l2^−/−^* embryos, we hypothesised that cells in this region do not segregate properly. To identify borders of prosomere 2 and its main subdivisions at molecular levels, we analysed the expression pattern of several diencephalic markers using *in situ* hybridisation. We used a *Tcf7l2* probe to stain the pretectum (prosomere 1) and prosomere 2, *Gbx2* probe to stain the glutamatergic thalamus, *Nkx2-2* and *Sox14* probes to stain the rostral thalamus and *Pax6* probe to stain the prethalamus (prosomere 3) and pretectum. These stainings confirmed that the thalamo-habenular area was fused and malformed in *Tcf7l2^−/−^* embryos (Fig. 4A,B). Also, we noticed that *Gbx2* staining was still present in the periventricular area, in which the youngest neurons are located, but absent in the intermediate and superficial portions of the thalamus (Fig. 4B), suggesting premature downregulation of *Gbx2*. The rostral thalamus area was elongated laterally (Fig. 4C). The boundary between prosomere 2 and 3, which was demarcated by the expression of *Pax6*, was disrupted (Fig. 4D).

To examine the boundaries at the cellular level, we stained brain sections with anti-PAX6, anti-SIX3 (marking the prethalamus), anti-NKX2-2 and anti-POU4F1 antibodies. Prosomere 2 cells were identified using anti-TCF7L2 (WT mice) or anti-β-galactosidase (KO mice) antibodies. In control embryos, the thalamic area was delineated rostro-ventrally by a narrow strip of PAX6-positive cells in a prethalamic subdomain (Fig. 4E), which separated the thalamus from the SIX3-positive prethalamic area (Fig. 4F), and the NKX2-2-positive area of the intergeniculate leaflet and ventral lateral geniculate nucleus did not overlap with the TCF7L2-high area of the caudal thalamus (Fig. 4G). Habenular cells were easily identified by POU4F1 staining, and the differences in cell densities distinguished the lateral from the medial part (Fig. 4H). In contrast, in *Tcf7l2* KO embryos, many PAX6-, SIX3-, NKX2-2- and POU4F1-positive cells were intermingled into the neighbouring thalamic territories (Fig. 4E-H). Sparse distribution of these cells, in particular POU4F1-positive cells, pointed to their unusual migration rather than identity switch. Consequently, the border between prosomere 2 and 3 was devoid of its sharpness and the thalamo-habenular border did not exist. This indicated that TCF7L2 plays a crucial role in the segregation of cells in subregional clusters in prosomere 2.

### Disrupted prosomere 2-specific regulatory network and altered expression of morphogenesis effector genes

**Fig. 5.**
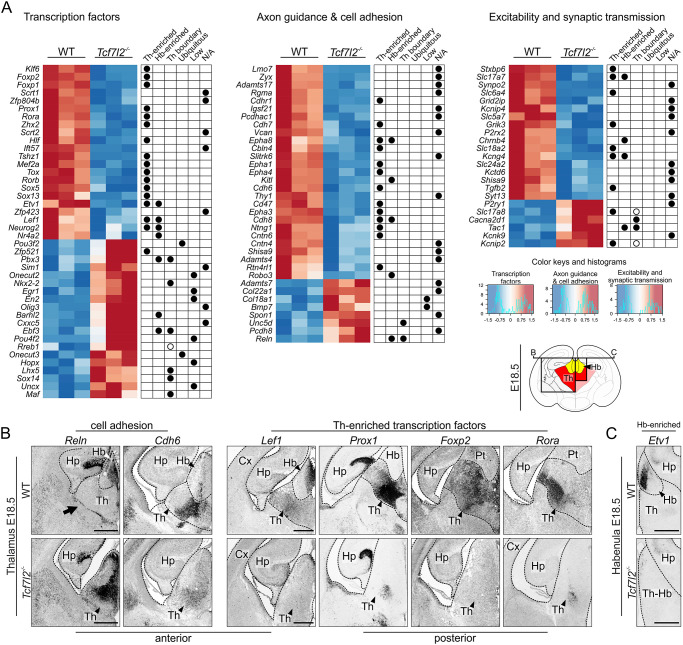
**TCF7L2 orchestrates genetic programme of morphological maturation in the thalamo-habenular region.** (A) Differentially expressed genes in the thalamo-habenular region in *Tcf7l2*^−/−^ embryos on E18.5 compared with WT embryos. Clustering heatmaps represent expression patterns of the genes from the overrepresented GO terms: transcription factors (left), axon guidance and cell adhesion molecules (centre) and genes related to neuron excitability and synaptic transmission (right). The log_2_ values of expression related to the median of the row are shown as a red-blue colour scale. Each line represents an independent biological replicate. Localisation of the expression of each gene in the brain, assessed in the Allen Brain Atlas, is marked with dots to the right of the matrices. Th-enriched, expression enriched in the thalamus; Hb-enriched, expression enriched in the habenula; Th boundary, expressed ubiquitously except for the thalamus (black dots) or expressed at the boundary of prosomere 2 (white dots); Ubiquitous, expressed throughout the brain; Low, overall low expression throughout the brain; N/A, not available in the Allen Brain Atlas. (B) *In situ* hybridisation with *Reln*, *Cdh6*, *Lef1*, *Prox1*, *Foxp2* and *Rora* probes in E18.5 coronal brain sections. A black arrow points to the border between the thalamus and prethalamus. (C) *In situ* hybridisation with *Etv1* probe in E18.5 coronal brain sections. Dotted lines demarcate different regions. Cx, cortex; Hb, habenula; Hp, hippocampus; Th, thalamus; Th-Hb, thalamo-habenular region. Scale bars: 0.5 mm.

To investigate the possible role of TCF7L2 in the regulation of a genetic programme of region-specific maturation in prosomere 2, we analysed global gene expression using RNA-seq in the thalamo-habenular region in WT and *Tcf7l2*^−/−^ embryos on E18.5. We found that 210 genes were significantly downregulated and 113 were upregulated in KO embryos by ≥0.4 or ≤−0.4 log_2_ fold-change (FC) (Fig. 5A, Table S1). Gene ontology (GO) term analysis of the differentially expressed genes (DEGs) revealed an overrepresentation of genes that are involved in transcription factor activity, anatomical structure development, neuron differentiation, axon guidance, cell adhesion, regulation of cell migration, regulation of transcription and synaptic signalling (Fig. 5A, Table S2). To determine whether the E18.5 DEGs from these groups are specific for prosomere 2, we inspected the corresponding *in situ* hybridisation images of brain sections in the Allen Brain Atlas (https://www.brain-map.org). In the selected groups, 100% of the downregulated genes were enriched in the thalamus, habenula, or both (Fig. 5A). Among them were prosomere 2-specific transcription factor genes, including known regulators of thalamic or habenular development *Rora*, *Foxp2*, *Etv1* and *Nr4a2* (Ebisu et al., 2016; Quina et al., 2009; Vitalis et al., 2017), cell adhesion molecules *Cdh6*, *Cdh8* and *Cntn6* (Bibollet-Bahena et al., 2017) and axon guidance genes such as *Epha4*, *Ntng1* and *Robo3* that encode important regulators of the guidance of thalamic or habenular efferent connections and the segregation of neurons in this region (Belle et al., 2014; Braisted et al., 2000; Dufour et al., 2003; Lehigh et al., 2013). Also, excitability genes, such as thalamus-enriched serotonin transporter gene *Slc6a4* that is expressed only during embryogenesis to regulate arborisation of thalamocortical axons (Chen et al., 2015), were downregulated in *Tcf7l2* KO embryos. In contrast, the list of the upregulated genes was dominated by the ones that were specifically expressed along thalamic borders or depleted from the thalamus, such as *Reln*, which is involved in neuronal migration and positioning (Hirota and Nakajima, 2017). An increased level of the rostral thalamus markers *Nkx2-2*, *Sox14* and *Lhx5* was also observed, and was likely caused by the expansion of this domain into the caudal thalamic area (Fig. 4C).

To confirm that the knockout of *Tcf7l2* caused mis-expression of prosomere 2-enriched or -depleted genes, we validated several of the identified E18.5 DEGs using *in situ* hybridisation. We observed strong ectopic expression of *Reln* and decreased expression of *Cdh6* in the thalamus in the mutant embryos (Fig. 5B). The expression of subregional markers of the caudal thalamus – the transcription factor genes *Lef1*, *Prox1, *Foxp2and* Rora* – was virtually absent in the thalamic area in *Tcf7l2^−/−^* embryos (Fig. 5B). Furthermore, *Tcf7l2* knockout abolished the expression of habenular markers *Lef1* (Fig. 5B) and *Etv1* (Fig. 5C) in different habenular subregions. These results revealed that TCF7L2 controls a network of prosomere 2 subregional transcription factors and regulates region-specific axon guidance and cell migration-related genes.

### Normal acquisition of glutamatergic identity but an impaired expression of postnatally induced synaptic and excitability genes in the thalamus

**Fig. 6.**
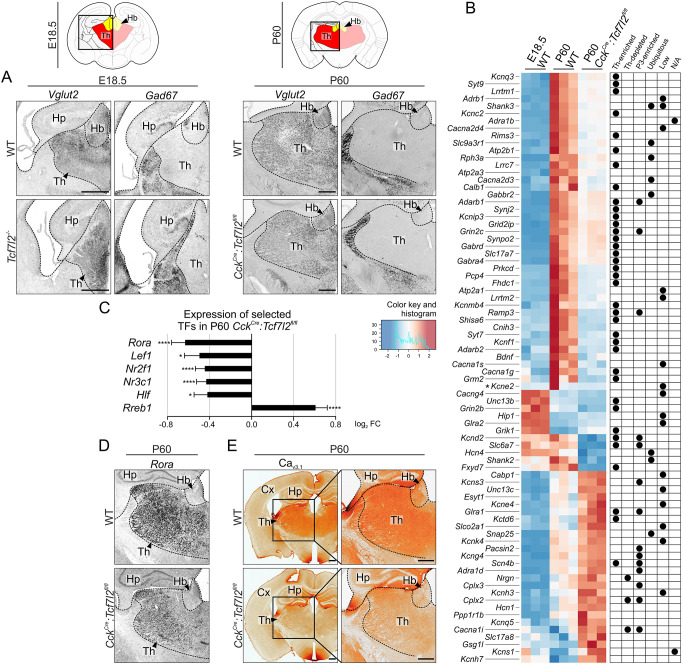
**TCF7L2 controls the expression of terminal excitability genes, but not VGLUT2 identity in the thalamus.** (A) *In situ* hybridisation with *Vglut2* and *Gad67* probes in E18.5 and P60 coronal brain sections. Schematic above indicates areas shown in micrographs below. (B) Differentially expressed genes in the thalamo-habenular region in *Cck^Cre^:Tcf7l2^fl/fl^* mice on P60 compared with control mice (WT). A clustering heatmap represents expression patterns of the genes from the overrepresented GO terms: ion-channels; neurotransmitter receptors and transmitters; G-proteins and synaptic vesicle proteins. The log_2_ values of expression related to the median of the row are shown as a red-blue colour scale. Each line represents an independent biological replicate. Localisation of the expression of each gene in the brain, assessed in the Allen Brain Atlas, is marked with black dots to the right of the matrices. Th-enriched, expression enriched in the thalamus; Th-depleted, expressed ubiquitously in the brain except for the thalamus; P3-enriched, enriched in prosomere 3; Ubiquitous, expressed throughout the brain; Low, overall low expression throughout the brain; N/A, not available in the Allen Brain Atlas. Asterisk next to *Kcne2* indicates that this gene is expressed specifically in the choroid plexus, so its high expression in one sample is most likely an artefact. (C) Differential expression of selected transcription factors in P60 *Cck^Cre^:Tcf7l2^fl/fl^* compared with control based on RNA-seq (Wald chi-squared test; *q≤0.05; ****q≤0.0001; error bars indicate standard error for log2 fold change). (D) *In situ* hybridisation with a *Rora* probe in P60 coronal brain sections. (E) DAB immunohistochemical staining of Ca_v3.1_ ion channel (encoded by *Cacna1g* gene) in P60 coronal brain sections. Dotted lines demarcate different regions. Magnification of boxed areas are shown as indicated. Cx, cortex; Hb, habenula; Hp, hippocampus; Th, thalamus. Scale bars: 0.5 mm.

We then asked whether TCF7L2 acts also as a terminal selector of thalamic phenotype, which is underlined by the expression of region-specific genes that determine neurotransmitter identity and electrophysiological characteristics. Habenular and thalamic neurons in rodents [except for GABAergic interneurons that are derived from the rostral thalamus (Evangelio et al., 2018)] are glutamatergic and express high levels of a vesicular glutamate transporter VGLUT2 (Fremeau et al., 2001; Herzog et al., 2001). To determine whether TCF7L2 is involved in the adoption of glutamatergic fate in the thalamus and habenula, we examined the expression patterns of *Vglut2* (also known as *Slc17a6*) and *Gad67* (also known as *Gad1*; a marker of GABAergic neurons) in the diencephalon in *Tcf7l2^−/−^* embryos on E18.5 and *Cck^Cre^:Tcf7l2^fl/fl^* P60 adult mice. Both knockout strains exhibited a pattern of GABAergic and glutamatergic cell distribution that was similar to the wild-type condition, with predominant *Vglut2* expression in prosomere 2 (Fig. 6A and Fig. S5A). Thus, TCF7L2 is not involved in the specification and maintenance of VGLUT2 identity in prosomere 2.

To investigate the hypothesis that TCF7L2 regulates terminal gene batteries, we compared global gene expression profiles in the thalamus between *Cck^Cre^:Tcf7l2^fl/fl^* and WT mice on P60 using RNA-seq. We found that 310 genes were significantly downregulated and 227 were upregulated in KO mice by ≥0.4 or ≤−0.4 log_2_ FC (Fig. 6B, Table S3). GO term enrichment analysis of the P60 DEGs revealed significant enrichment with terms that clustered into groups of synaptic proteins and regulators of membrane conductance: regulation of ion transport, voltage-gated channel activity, regulation of membrane potential, G-protein coupled receptor signalling pathway, regulation of trans-synaptic signalling and regulation of synapse organisation (Fig. 6B, Table S4). Transcription factor genes were not overrepresented in the P60 DEGs. However, six thalamus-enriched subregional transcription factor genes were significantly downregulated or upregulated in *Cck^Cre^:Tcf7l2^fl/fl^* mice on P60 (Fig. 6C), including *Rreb1* that is expressed in the thalamus postnatally, *Lef1* and *Rora*, the latter confirmed by *in situ* hybridisation (Fig. 6D and Fig. S5B).

To investigate whether TCF7L2 regulates thalamus-specific or generic neuronal features, we examined spatial expression profiles of the identified excitability/synaptic genes in this cluster in the Allen Brain Atlas. The vast majority of the downregulated genes are expressed specifically in the thalamus (Fig. 6B), for example *Kcnc2* and *Cacna1g*, which encode subunits of K_v3.2_ voltage-gated potassium channels and Ca_v3.1_ voltage-gated calcium channels, respectively (Kasten et al., 2007; Kim et al., 2001). The downregulation of Ca_v3.1_ was further confirmed using immunohistochemistry (Fig. 6E and Fig. S5C). Also, the habenular and thalamic glutamate transporter gene *Vglut1* was downregulated, but not *Vglut2* that encodes the main thalamic glutamate transporter, consistently with the *in situ* hybridisation results (Fig. 6A and Fig. S5A). Conversely, only a few of the upregulated excitability/synaptic genes were thalamus-enriched.

To investigate the hypothesis that TCF7L2 regulates a genetic programme of terminal selection that is activated postnatally, we crossed the selected group of genes with a list of genes that were differentially expressed between E18.5 and P60 in WT mice. Almost 90% of the synaptic/excitability P60 DEGs that were thalamus-enriched were induced after embryogenesis, confirming that TCF7L2 functions as a terminal selector during postnatal development (Fig. 6B). Thus, postnatally, TCF7L2 only partly regulates the expression of thalamic transcription factors, but controls a battery of genes that are induced postnatally and shape terminal electrophysiological identities of thalamic neurons.

### Direct regulation of thalamic terminal effector genes by TCF7L2

**Fig. 7.**
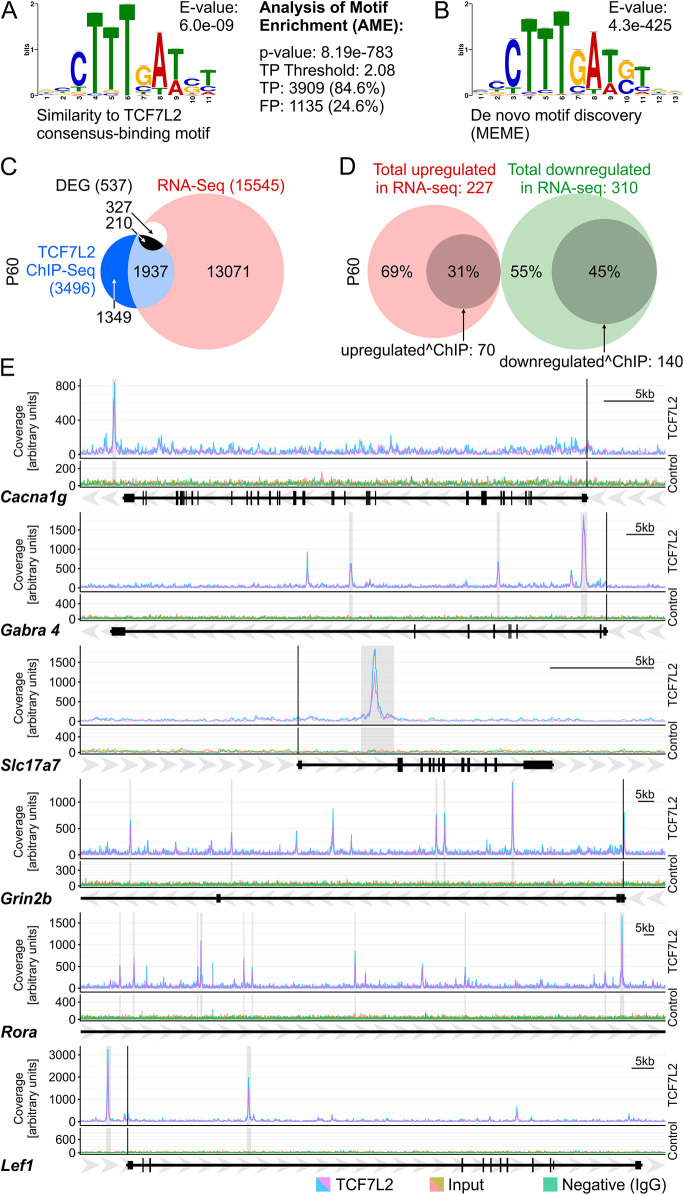
**TCF7L2 directly regulates terminal effector genes in the thalamus.** (A) Enrichment analysis of the TCF7L2 consensus motif in the TCF7L2 ChIP peaks by the AME algorithm. TP, true positive peaks, where the TCF7L2 motif was identified; FP, false positive peaks, where the TCF7L2 motif was identified in reshuffled peaks data. (B) *De novo* motif discovery analysis. The most significant motif identified by MEME-ChIP is TCF7L2. (C) Venn diagram showing the overlap between genes identified by RNA-seq (red) and ChIP-seq (blue). The P60 DEGs are in white, DEGs overlapping with ChIP-seq are in black. (D) Venn diagrams showing the overlap between the upregulated (red) or downregulated (green) P60 DEGs and genes identified by ChIP-seq (grey). (E) TCF7L2 binding profiles in the *Cacna1g*, *Gabra4*, *Slc17a7* (*Vglut1*), *Grin2b*, *Rora* and *Lef1* genes. Two independent biological replicates are represented as separate lines: pink/blue, the TCF7L2 ChIP-seq signal; orange/olive, input; green, negative control (IgG).

To understand how TCF7L2 regulates terminal effector genes in the thalamus, we performed a ChIP-seq analysis on the thalami isolated from adult WT mice on P60. We used the same antibody that we used for the western blots and immunofluorescence/immunohistochemistry in this study, and which was validated with samples from the mutant animals (Fig. 2B-G). This antibody was previously used in ChIP-seq by other authors on different cell types (Frietze et al., 2012; Geoghegan et al., 2019; Norton et al., 2011). Analysis resulted in 4625 peaks in the anti-TCF7L2 precipitated samples with fold enrichment (FE) ≥10 over input sample, which annotated to 3496 unique genes (Table S5). Analysis of motif enrichment (the AME algorithm from the MEME suite) showed significant overrepresentation of the consensus motif for TCF7L2 in the sequences bound by the anti-TCF7L2 antibody. This motif was detected in almost 85% of the TCF7L2 ChIP-seq peaks (Fig. 7A), validating the experiment. In addition, we validated the experiment by using a thalamic sample from *Cck^Cre^:Tcf7l2^fl/fl^* mice. We observed that 94.3% of the peaks identified in the WT condition were not detected in this sample, proving specific target recognition in our assay.

To find the most frequent motifs in the TCF7L2 ChIP-seq peaks, we performed *de-novo* motif discovery using MEME-ChIP – 17 motifs were identified. The most significantly overrepresented motif was identical to the TCF7L2/TCF7L1 consensus binding site (*E*-value=4.3e^−425^; Fig. 7B). The motifs of GCR (NR3C1), RREB1 and RORA were also overrepresented (*E*-value=1.3^−32^, 2.3^−30^, 2.2^−23^, respectively; Fig. S6A). These transcription factors are enriched in the thalamus, their expression was altered in *Cck^Cre^:Tcf7l2^fl/fl^* mice, and their genes were identified by the ChIP-seq, suggesting that not only are they downstream targets of TCF7L2, but they also cooperate with TCF7L2 in gene expression regulation.

The peaks annotated to genes that were expressed in the thalamus on P60 were most frequently localised in intronic regions that may act as intragenic enhancers (Fig. S6B and Table S5). The remaining peaks (i.e. annotated to non-expressed genes) were mainly annotated to predicted genes and pseudogenes, and were located in distal intergenic regions, suggesting that they represent distal enhancers of unidentified genes. The DEGs with no annotated ChIP-seq peaks can be indirect targets of TCF7L2 or regulated by TCF7L2-dependent distal enhancers.

GO term enrichment analysis performed on genes significantly bound by TCF7L2 and expressed at P60 revealed an overrepresentation of genes related to the regulation of membrane potential and synaptic signalling, calcium and potassium ions transmembrane transport, and the regulation of neuron projection development and cell adhesion (Table S6). Moreover, genes bound by TCF7L2 were highly overrepresented in the P60 DEGs (Fig. 7C). The ChIP-seq peaks were detected in 31% genes that were upregulated and in 45% genes that were downregulated, including many thalamus-enriched genes that are involved in synaptic signalling or membrane excitability (Fig. 7D,E and Table S5). These genes were either broadly expressed in the thalamus (such as *Cacna1g*, *Gabrd*, *Kcnc2*, *Syt7*, *Gabra4*, *Grm1*, *Grid2ip* or *Synpo2*) or restricted to thalamic subregions (such as *Grm1* to the anterodorsal and mediodorsal nuclei, *Cacng3* to the PV and midline nuclei, *Kcnab2* to the PF, AD and ventral nuclei, and *Kcnd2* to the AD, PV and habenula). This confirmed that TCF7L2 is directly involved in the activation of genes that define pan-thalamic terminal identity and subregional identities in the thalamus.

### Severe impairments in excitability of thalamic neurons in adult mice

**Fig. 8.**
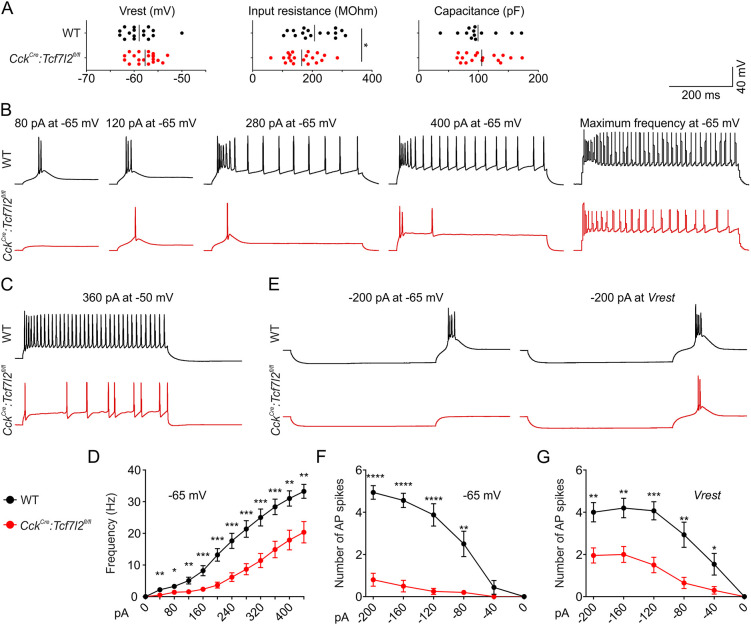
**TCF7L2 is essential postnatally for the development of burst and tonic firing patterns in thalamocortical relay neurons.** (A) Basic electrical properties of cell membrane measured in VB neurons in brain slices: input resistance (WT *n*=16, *Cck^Cre^:Tcf7l2^fl/fl^ n*=20; two-tailed unpaired Student's *t*-test; *P*-value=0.0443); resting potential (WT *n*=16, *Cck^Cre^:Tcf7l2^fl/fl^ n*=20; Mann–Whitney test; *P*-value=0.1112); membrane capacitance (WT *n*=13, *Cck^Cre^:Tcf7l2^fl/fl^ n*=17; Mann–Whitney test; *P*-value=0.6725). (B,C) Representative traces from whole-cell patch-clamp recordings from VB neurons in brain slices at −65 mV membrane potential and increasing depolarising current inputs (burst and tonic spikes) (B), and at −50 mV membrane potential and depolarising current inputs (tonic spikes) (C). (D) Frequency of spikes evoked by increasing depolarising currents at −65 mV, as represented in B (WT *n*=16, *Cck^Cre^:Tcf7l2^fl/fl^ n*=20; Mann–Whitney test). (E) Representative traces from whole-cell patch-clamp recordings from VB neurons in brain slices at −65 mV or the resting membrane potential (*Vrest*, ∼−57 mV) and hyperpolarising current inputs (rebound bursts of spikes). (F,G) The number of spikes per rebound burst at −65 mV (F) (WT *n*=16, *Cck^Cre^:Tcf7l2^fl/fl^ n*=20; Mann–Whitney test) or at the resting membrane potential (*Vrest*) (G) (WT *n*=15, *Cck^Cre^:Tcf7l2^fl/fl^ n*=20; Mann–Whitney test), as represented in E. *P≤0.05, **P≤0.01, ***P≤0.001, ****P≤0.0001; error bars in D,F and G indicate s.e.m.

To functionally test the role of TCF7l2 in the regulation of thalamic neuron electrophysiological properties, we used whole-cell patch-clamp recordings in *Cck^Cre^:Tcf7l2^fl/fl^* mice from targeted thalamocortical neurons in the VB. We first tested the basic properties of VB neurons. Resting potential and capacitance were similar in WT and KO mice, but input resistance significantly decreased in KO mice (Fig. 8A). We then examined whether the targeted neurons were able to evoke action potentials (APs; e.g. tonic, burst and rebound burst modes) that are typical for thalamocortical cells. We used depolarising current steps to evoke both sustained trains of APs (tonic) and burst firing at the beginning of a train (Fig. 8B,C). Thalamic cells produced fewer APs in *Cck^Cre^:Tcf7l2^fl/fl^* mice in both tonic and burst firing modes (Fig. 8D). Rebound bursts, which are crucial for the response of thalamocortical neurons to inhibitory input, were evoked by steps of hyperpolarising current (Fig. 8E). Most neurons from *Tcf7l2* KO mice did not show any rebound bursts at the hyperpolarising membrane potential (−65 mV; Fig. 8F). Hyperpolarising steps that were applied at the resting membrane potential (∼−57 mV) evoked rebound burst spiking in VB neurons in *Cck^Cre^:Tcf7l2^fl/fl^* mice, but the number of spikes was approximately half that seen in WT mice (Fig. 8G). These dramatic impairments in electrophysiological responses demonstrated that TCF7L2 is essential for the establishment of unique excitability and firing patterns in thalamocortical neurons.

## DISCUSSION

Little is understood about how the lengthy process of postmitotic differentiation is regulated in the vertebrate brain. The present study identifies TCF7L2 as a master regulator of regional transcription factors in the thalamus and habenula, and a selector of stage-specific developmental programmes that switch postnatally from morphological to electrophysiological maturation.

### TCF7L2 and a network of transcription factors regulate morphological maturation of the thalamus and habenula

TCF7L2 is the only developmentally regulated transcription factor that is expressed postmitotically throughout prosomere 2 (Nagalski et al., 2016). TCF7L2 is not necessary for the induction of caudal thalamic and pan-habenular identities that are defined by *Gbx2* and *Pou4f1* expression, respectively, because the expression of these markers was not abolished in *Tcf7l2* KO embryos during neurogenesis (E12.5). The maintenance of *Gbx2* expression in the intermediate and superficial thalamic portions may depend on TCF7L2 at later stages, because *Gbx2* staining in the mutant thalami persisted only in the periventricular area, in which the youngest neurons are located. An apparent increase in the number of POU4F1 cells in *Tcf7l2*^−/−^ embryos on E12.5 suggests that TCF7L2 could play a regulatory role in cross-repressing thalamic and habenular identities, by promoting thalamic fate, in agreement with a previous conclusion (Lee et al., 2017). However, decreased expression of sub-habenular markers, e.g. *Etv1* and *Nr4a2*, as well as a thalamo-habenular marker *Lef1*, and sub-thalamic markers, e.g. *Foxp2*, *Prox1* and *Rora* in *Tcf7l2*^−/−^ embryos on E18.5 indicates that TCF7L2 plays a positive role in the development and diversification of both thalamic and habenular identities. *Tcf7l2* knockout did not inhibit the expression of rostral thalamic markers, *Nkx2-2*, *Sox14* and *Lhx5*, indicating a different role of TCF7L2 in this particular subdomain of prosomere 2.

A direct regulation of the above subregional markers by TCF7L2 is supported by our previous *in vitro* results showing that TCF7L2 and the closely related factor LEF1 can regulate promoters of thalamic factors *Gbx2*, *Foxp2* and *Rora* (the latter one confirmed in the present study by our ChIP-seq on P60), and habenular factors *Pou4f1*, *Nr4a2* and *Etv1* (Nagalski et al., 2016). The mechanism of differential regulation of genes by TCF7L2 in subregions of prosomere 2 is not known. A differentiating factor might be the level of TCF7L2, which varies between thalamic nuclei during embryogenesis. Another possibility is cooperation with independently induced transcription factors that are restricted to smaller areas in prosomere 2. GBX2 is a good candidate, considering that *Foxp2* and *Rora* were also downregulated in *Gbx2*^−/−^ embryos (Mallika et al., 2015). We speculate that different levels of GBX2, expression of which decreases in the developing thalamus in the latero-medial gradient (Li et al., 2012), might contribute to subregional diversification of thalamic identities by TCF7L2. Striking similarities between phenotypes in *Tcf7l2*^−/−^ and *Gbx2*^−/−^ embryos, such as the elongated shape of the thalamo-habenular region, impaired cell segregation and the absence of thalamocortical projections (Chatterjee et al., 2012; Chen et al., 2009), corroborates the conclusion that TCF7L2 and GBX2 cooperate in the thalamus during embryogenesis. Less severe impairments in the establishment of thalamocortical connections were reported in *Foxp2* (Ebisu et al., 2016) and *Rora* (Vitalis et al., 2017) KO mice, and mice with either knockout exhibited molecular identity impairments that were restricted to subregions of the thalamus. This implies that postmitotic differentiation in the thalamus is regulated by a hierarchical network of regional and subregional transcription factors.

Similarities also exists between *Tcf7l2^−/−^* and *Pou4f1*^−/−^ embryos. Subregional habenular markers *Etv1* and *Nr4a2* were also downregulated in *Pou4f1*^−/−^ embryos (Quina et al., 2009), and the expression of habenular axon guidance genes *Rgma* and *Epha8* decreased in *Pou4f1*^−/−^ as well as *Tcf7l2^−/−^* embryos (Quina et al., 2009). However, a comparison between the effects of *Tcf7l2* and *Pou4f1* knockouts in the habenula is not straightforward. Anatomical impairments were much more severe in *Tcf7l2*^−/−^ embryos, but much of this phenotype may be attributed to secondary effects that result from the spread of POU4F1-positive cells throughout the lateral part of prosomere 2 in *Tcf7l2*^−/−^ embryos.

Presumably, cell non-autonomous and secondary mechanisms contribute to morphological malformation of the thalamo-habenular region. Considering that PAX6-positive prethalamic cells do not express *Tcf7l2* in WT embryos, abnormal intermingling of these cells into thalamic territory must be cell non-autonomous. The same may apply to the impaired segregation of rostral thalamic and habenular cells. Mechanisms that regulate cell migration and nucleogenesis in the diencephalon are poorly understood. We speculate that mis-expression of cell adhesion genes in the thalamus, such as ectopic expression of *Reln* and decreased expression of thalamus-specific genes *Cdh6*, *Cdh8* and *Cntn6*, could turn the thalamus into a permissive environment for cells migrating from the neighbouring *Reln*-positive structures, i.e. prethalamus, rostral thalamus, habenula and, possibly, pretectum. Considering that topographic axonal connections can create physical boundaries in the developing brain, disorganised stria medullaris or afferent connections from the retina, pretectum and midbrain, where *Tcf7l2* is expressed at high levels (Nagalski et al., 2013; Vacik et al., 2011), may also play a role.

Previous research has shown that the aberrant growth of thalamocortical axons toward the hypothalamus instead of the ventral telencephalon in *Tcf7l2*^−/−^ embryos resulted from unresponsiveness of thalamic cells to Slit-repulsive ligands, due to decreased expression of genes that encode Slit receptors Robo1 and Robo2 (Lee et al., 2017). We did not observe any changes in the levels of *Robo1* and *Robo2* mRNA on E18.5. The expression of these genes is specific for prosomere 2 only at earlier stages (https://developingmouse.brain-map.org); hence it may not depend on TCF7L2 at late gestation. Instead, we observed decreased expression of genes that encode habenular axon-navigating molecules ROBO3 and RGMA and thalamic axon-navigating molecules that are later induced and subregion-specific, e.g. NTNG1, EPHA1/3/4/8. EPHA4 regulates topographical sorting of VB axons in the ventral telencephalon at late gestation (Dufour et al., 2003). This implicates TCF7L2 in controlling the sequential steps of thalamocortical axon navigation and subregional sorting.

### TCF7L2 controls the acquisition of characteristic excitability patterns in the thalamus

Many genes that were downregulated in mice with the postnatal knockout of *Tcf7l2* and/or identified as direct TCF7L2 targets using the ChIP-seq assay encode postnatally-induced and thalamus-enriched proteins involved in neural signal transmission. The examples are voltage-gated T-type Ca_v3.1_ calcium channels and K_v3.2_ potassium channels (encoded by thalamus-enriched *Cacna1g* and *Kcnc2*, respectively). This is consistent with our previous *in silico* predictions and ChIP-qPCR, which showed that β-catenin, which is a cofactor of LEF1/TCF transcription factors, interacts with promoters of several excitability/synaptic genes, including, in particular, *Cacna1g* (Wisniewska et al., 2010, 2012).

Excitability and synaptic transmission parameters are specific to different classes of neurons and together ensure the proper functioning of neural circuits. Functional experiments in the present study demonstrate that thalamocortical relay neurons lose their proper tonic and burst firing patterns in the absence of TCF7L2. The downregulation of *Cacna1g* and *Kcnc2* most likely contributed to this phenotype, given that *Cacna1g* knockout resulted with the absence of burst firing (Kim et al., 2001), whereas K_v3.2_ channel inhibition suppressed the firing rate in tonic mode (Kasten et al., 2007) in thalamocortical neurons, similar to *Tcf7l2* knockout. These results implicate TCF7L2 in the regulation of postnatal genes that control thalamic terminal excitability patterns.

The thalamus is molecularly distinguishable from other brain structures, but many thalamus-enriched genes are differentially expressed between thalamic nuclei or groups of nuclei (Nagalski et al., 2016; Phillips et al., 2019). TCF7L2 was proved to regulate genes that are broadly expressed in the thalamus and those that are specifically expressed in groups of thalamic nuclei. *Tcf7l2* was not knocked out in PV and PF, and was less efficiently knocked out in the AD or midline nuclei, preventing us from investigating the involvement of TCF7L2 in gene expression regulation in these regions. Nonetheless, ChIP-seq analysis identified TCF7L2 peaks in excitability/synaptic genes, the expression of which is enriched specifically in PV, PF or AD, implicating TCF7L2 in the direct control of subregional as well as pan-regional terminal selection in the thalamus. Cooperation with subregional thalamic transcription factors, such as RORA, NR3C1 and RREB1, as suggested by the overrepresentation of the corresponding binding motifs in the TCF7L2 ChIP-seq peaks, could contribute to TCF7L2-dependent regulation of differentially expression thalamic genes, but this needs further investigation.

### TCF7L2 is a terminal selector in the thalamus

TCF7L2 meets the criteria of a thalamic terminal selector: its expression is induced during neurogenesis and maintained in thalamic neurons throughout life (Nagalski et al., 2013), it directly binds to thalamic terminal differentiation genes, and its activity is required for electrophysiological maturation of the thalamus. Besides, TCF7L2 orchestrates morphological maturation of the thalamo-habenular region during embryogenesis. This double function differentiates TCF7L2 from the majority of other known terminal selectors, which play minor roles in cell migration or axon guidance (Hobert, 2016), for example habenular selector POU4F2 (Serrano-Saiz et al., 2018). Other examples of terminal selectors that regulate axon guidance are the glutamatergic selector of corticospinal neurons FEZF2 and serotoninergic selector PET1 (FEV) (Donovan et al., 2019; Lodato et al., 2014).

TCF7L2 is essential for the postnatal induction of terminal functional properties of thalamic neurons. However, unlike classic terminal selectors, TCF7L2 does not control neurotransmitter identity, which in the thalamus depends on VGLUT2. Recent research reported that cells in a thin superficial portion of the thalamus switched to GABAergic identity in *Tcf7l2^−/−^* embryos, shown by the colocalisation of *Gad67* and *Gbx2*-driven tdTomato signal (Tran et al., 2020). However, the staining resolution does not allow a conclusion that the signals colocalised in the same cells; and according to the most recent research the origin of thalamic GABAergic cells may be assigned to prethalamic, rostral thalamic and even pretectal domains (Jager et al., 2016; Puelles et al., 2020). More importantly, a normal pattern of *Vglut2* and *Gad67* expression in mice with the postnatally induced knockout of *Tcf7l2* demonstrates that TCF7L2 does not play a role in maintaining glutamatergic identity in thalamic neurons. This implies that, in the case of glutamatergic neurons in the thalamus, the selection of different terminal features is uncoupled. It is known from studies of invertebrate and vertebrate neurons that the adoption of neurotransmitter identity and other terminal features is often linked (Serrano-Saiz et al., 2013). For example, in contrast to thalamic cells and TCF7L2, PET1 controls the early postmitotic adoption of neurotransmitter phenotype and the postnatal acquisition of excitability features by serotonergic cells in the raphe nucleus (Hendricks et al., 2003; Liu et al., 2010; Wyler et al., 2016), and neurotransmitter metabolism genes together with ion channel genes constitute a co-varying module in midbrain dopaminergic neurons (Tapia et al., 2018). Furthermore, studies on glutamatergic neurons in mice concluded that POU4F1 and FEZF2 are selectors of VGLUT1 identity in the medial habenula and corticospinal neurons, respectively, as well as regulators of many other neuron subtype–specific genes (Chen et al., 2008; Lodato et al., 2014; Serrano-Saiz et al., 2018). There are only a few examples of separate regulation of neurotransmitter identity and other terminal features. In *Caenorhabditis elegans*, *unc-3* regulates cholinergic identity in command interneurons, but not other terminal features (Pereira et al., 2015), and *ttx-3* controls the terminal differentiation of neurosecretory-motor neurons, but not their serotoninergic identity (Zhang et al., 2014). More research is needed to build models of the postmitotic regulation of neurons in vertebrates and compare regulatory strategies between vertebrates and invertebrates.

### Conclusion

The present study sheds new light on vertebrate regulatory strategies in the postmitotic differentiation of molecularly diverse neurons that share a glutamatergic identity. We found that temporarily separated developmental events and molecular diversification of neurons within a region can be controlled by a single regional transcription factor, as exemplified by TCF7L2 and prosomere 2. Finally, we showed that electrophysiological maturation can be uncoupled from the selection of neurotransmitter identity. Considering that *Tcf7l2* is associated with mental disorders, our findings also provide a new insight into the aetiology of thalamic and habenular dysfunction that are observed in these disorders.

## MATERIALS AND METHODS

### Animals

We used C57BL/6NTac-*Tcf7l2*^tm1a^ (EUCOMM)Wtsi/WtsiIeg (*Tcf7l2*^tm1a^) mouse strain (Skarnes et al., 2011), with a trap cassette upstream of the critical exon 6 of the *Tcf7l2* gene. To generate the *Cck*^Cre^*:Tcf7l2*^fl/fl^ strain, in which the knockout of *Tcf7l2* is induced in the thalamic area perinatally (http://connectivity.brain-map.org/transgenic/imageseries/list/1.html?gene_term=Cck-IRES-Cre), *Tcf7l2*^tm1a/+^ animals were first crossed with flippase-expressing mice (ROSA26::FLPe knock in strain; JAX stock #009086, The Jackson Laboratory; Farley et al., 2000), and then with *Cck*^tm1.1(cre)Zjh^/J mice (*Cck*^Cre^*:Tcf7l2*^+/+^, JAX stock #012706, The Jackson Laboratory; Taniguchi et al., 2011), which express Cre recombinase from the *Cck* promoter. *Cck^Cre^:Tcf7l2^+/+^* animals were used as wild type. To generate the *Cck^Cre^:tdTomato^fl/+^* reporter strain, *Cck^Cre^:Tcf7l2^+/+^* mice were crossed with the homozygous Ai9(RCL-tdT) strain. Mice were maintained on a 12 h/12 h light/dark cycle with *ad libitum* access to food and water. For the experimental procedures all mice were selected by PCR-based genotyping: Tcf7l2^tm1a^ and Tcf7l2^fl^ alleles: tcf_F, GGAGAGAGACGGGGTTTGTG; tcf_R, CCCACCTTTGAATGGGAGAC; floxed_PNF, ATCCGGGGGTACCGCGTCGAG; Tm1c_R, CCGCCTACTGCGACTATAGAGA; Cck^Cre^ allele: 11214, GAGGGGTCGTATGTGTGGTT; 11215, GGGAGGCAGATAGGATCACA; 9989, TGGTTTGTCCAAACTCATCAA. All of the experimental procedures were conducted in compliance with the current normative standards of the European Community (86/609/EEC) and the Polish Government (Dz.U. 2015 poz. 266). Animal usage was controlled by the institutional advisory board for animal welfare at the Centre of New Technologies.

### Brain fixation and brain slice preparation

Embryos were collected on E12.5 or E18.5. Noon on the day of appearance of the vaginal plug was considered E0.5. Timed-pregnant dams were sacrificed by cervical dislocation, the embryos were removed and decapitated. E18.5 brains were dissected out and fixed overnight in 4% paraformaldehyde (PFA; P6148, Sigma-Aldrich) in 0.1 M phosphate-buffered saline (pH 7.4) (PBS; PBS404, BioShop) at 4°C. E12.5 heads were fixed whole. Adult mice were sacrificed on P60-P75 (further referred to as P60) by pentobarbital sedation and perfusion with PBS and 4% PFA. Their brains were dissected out and fixed overnight in 4% PFA. For cryostat sections, the brains were sequentially transferred into 15% and 30% sucrose in PBS at 4°C until they sank. Next, E12.5 and E18.5 tissues were embedded in 10% gelatine/10% sucrose solution in PBS (Ferran et al., 2015a,b). Postnatal brains were transferred to O.C.T. (4583, Sakura Tissue-Tek). Tissues were frozen in −60°C isopentane. Sections (embryos, 20 μm; adult, 40 μm) were obtained using a Leica CM1860 cryostat. Embryonic sections were mounted directly on SuperFrost-plus slides (J1800AMNZ, Menzel-Gläser). Adult tissue was collected as free-floating sections into an anti-freeze solution (30% sucrose/30% glycerol in PBS). For DiI axon tracing, E18.5 immersion-fixed brains were kept in 4% PFA at 4°C. We used 3-5 embryos/mice per genotype from at least two litters in each analysis by Nissl staining, DiI axon tracing, immunohistochemistry or *in situ* hybridisation. Stained sections were visualised under a Nikon Eclipse Ni-U microscope.

### Nissl staining

Brain sections were dehydrated in an ethanol series (50%, 70%, 95% and 99.8%), cleared in xylene and rehydrated. The sections were rinsed in tap water and stained with 0.13% (w/v) Cresyl Violet solution (CS202101, Millipore) for 4 min, rinsed and dehydrated again as described above. The slices were washed in xylene and mounted using EuKitt.

### Chromogenic *in situ* hybridisation

*In situ* hybridisation was performed in cryosections as previously described (Ferran et al., 2015b; Puelles et al., 2016), using digoxigenin-UTP-labelled sense (not shown) and antisense riboprobes, synthesised with the DIG RNA labelling kit (11175025910, Roche). Plasmids for the synthesis of *Cdh6*, *Foxp2*, *Gad67*, *Gbx2*, *Lef1*, *Prox1*, *Rora*, *Tcf7l2* and *Vglut2* probes come from the collection of José Luis Ferran; the other plasmids were kind gifts from: James Li from the Department of Genetics and Developmental Biology, University of Connecticut Health Center, Farmington, CT, USA (*Etv1*) (Chatterjee et al., 2014); Seth Blackshaw from the Johns Hopkins University School of Medicine, Baltimore, MD, USA (*Nkx2-2* and *Sox14*) (Shimogori et al., 2010); Tomomi Shimogori from the RIKEN Center for Brain Science, RIKEN, Saitama, Japan (*Reln*) (Chiara et al., 2012); David Price from the Centre for Integrative Physiology, University of Edinburgh, UK (*Pax6*) (Walther and Gruss, 1991). The *Tcf7l2* probe spans the first eight exons of the *Tcf7l2* gene, therefore it detects also *Tcf7l2* transcripts which are truncated after exon 5 in the mutant mice. The stained sections were washed in xylene and mounted using EuKitt (03989, Sigma-Aldrich).

### Fluorescent immunohistochemistry

Frozen sections were washed in PBS with 0.2% Triton X-100 (PBST) and blocked with 5% normal donkey serum (NDS) for 1 h. The slides were then incubated with primary antibodies mixed in 1% NDS overnight at 4°C. Antibodies against TCF7L2 (1:500; 2569, Cell Signaling Technology), Ca_v3.1_ (1:500; MABN464, Sigma-Aldrich, NeuroMab clone N178A/9), β-galactosidase (1:100; AB986, Merck Millipore), L1CAM (1:500; MAB5272, Merck Millipore), PAX6 (1:100; PRB-278P, BioLegend), KI-67 (1:100; AB9260, Merck Millipore), TUJ1 (1:65; MAB1637, Merck Millipore), NKX2-2 (1:50; 74.5A5, Developmental Studies Hybridoma Bank), SIX3 (1:100; 200-201-A26S, Rockland), POU4F1 (1:300; Fedtsova and Turner, 1995) were used. Sections were then incubated for 1 h with appropriate secondary antibody conjugated with Alexa Fluor 488 or 594 (1:500; A-21202, A-21207 and A-11076, Thermo Fisher Scientific). The slides were additionally stained with Hoechst 33342 (1:10,000; 62249, Thermo Fisher Scientific), washed and mounted with Vectashield Antifade Mounting Medium (H1000, Vector Laboratories).

### DAB immunohistochemistry

Free-floating sections were washed in PBST, incubated in 0.3% H_2_O_2_ for 10 min, blocked with 3% normal goat serum (NGS) for 1 h and incubated with primary antibodies against TCF7L2 (1:1000) or Ca_v3.1_ (1:500) in 1% NGS overnight at 4°C. Next, sections were incubated for 1 h with biotinylated goat anti-rabbit antibody (1:500 BA-1000, Vector Laboratories) or horse anti-mouse antibody (1:500, BA-2000, Vector Laboratories) in 1% NGS, and then for 1 h in Vectastain ABC reagent (PK-6100, Vector Laboratories). Staining was developed using 0.05% DAB (D12384, Sigma-Aldrich) and 0.01% H_2_O_2_. Next, sections were mounted onto SuperFrost Plus slides, dehydrated in an ethanol series (50%, 70%, 95% and 99.8%), washed in xylene and mounted using EuKitt.

### DiI axon tracing

PFA-fixed brains were separated into hemispheres and small DiI crystals (D-3911, Thermo Fisher Scientific) were placed in the exposed thalamic surface. Tissue was then incubated in 4% PFA at 37°C for 18-21 days. The hemispheres were then embedded in 5% low-melting-point agarose and cut into 100 μm thick coronal sections using a vibratome. The sections were counterstained with Hoechst, mounted onto glass slides and secured under a coverslip with Vectashield Antifade Mounting Medium.

### Western blot analysis

Protein extracts were obtained from six animals per genotype from at least two litters. The thalamo-habenular regions were dissected from the brains (Fig. S7A,B) and homogenised in ice-cold RIPA buffer. Protein concentrations were determined using the Bio-Rad protein assay (5000006, Bio-Rad Laboratories). Clarified protein homogenate (50 μg) was loaded on 10% SDS-polyacrylamide gels. Separated proteins were then transferred to Immun-Blot PVDF membranes (1620177, Bio-Rad Laboratories), which were then blotted with anti-TCF7L2 (1:1000; 2569, Cell Signaling Technology), anti-β-actin (1:500; A3854, Sigma-Aldrich) and anti-GAPDH (1:1000; SC-25778, Santa Cruz Biotechnology) antibodies. The staining was visualised with peroxidase substrate for enhanced chemiluminescence (ECL) and 200 μM coumaric acid. Images were captured using the Amersham Imager 600 RGB (General Electric).

### Quantification of Ki-61

Ki-67-positive cells were counted manually in the prosomere 2 region in E12.5 brain sections from control and *Tcf7l2^−/−^* animals from three different litters (three mice, four sections each per genotype). Areas of each prosomere 2 section were measured using ImageJ and the number of Ki-67-positive cells /1 mm^2^ was calculated. Two-tailed unpaired Student's *t*-test was used to test for the difference between two groups.

### RNA isolation and RNA-seq analysis

Mice were collected on E18.5 and P60. The thalamo-habenular regions were dissected-out immediately (Fig. S7A,B), and the RNA was extracted using QIAzol (79306, Qiagen) and the RNeasyMini Kit (74106, Qiagen). The quality of RNA was verified using Bioanalyzer (Agilent). RNA samples from three animals (two litters) for each genotype were sequenced on the same run of Illumina HiSeq2500. The reads were aligned to the mouse genome mm10 assembly from the University of California, Santa Cruz (UCSC) using HISAT (Kim et al., 2015) and their counts were generated using HTSeq (Anders et al., 2015). Differential gene expression analysis was performed with DeSeq2 (Love et al., 2014). Genes with log_2_(FC)≥0.4 and log_2_(FC)≤−0.4 and FDR adjusted *P*-value (*q*-value)≤0.05 were considered to be the differentially expressed up- and downregulated genes.

### Functional enrichment analysis

Gene ontology enrichment was performed using an open access online tool GOrilla (http://cbl-gorilla.cs.technion.ac.il/). Two unranked lists of genes, one target and one universal (all detected transcripts) were used, assuming a hypergeometric distribution of the unranked genes. GO-Term enrichments were tested with Fisher's exact test, and FDR adjusted *P*-values (*q*-value)≤0.01 were considered significant.

### ChIP-seq

Mice were collected on P60 and sacrificed by cervical dislocation. The thalami were dissected-out (Fig. S7B), chopped and fixed for 10 min in 1% formaldehyde (114321734, Chempur), followed by 10 min of quenching with stop solution (53040, Activ Motif). Chromatin was isolated from cellular nuclei (Cotney and Noonan, 2015), and then sonicated into 200-500 bp fragments with Covaris S220. Chromatin from six mice (two litters) was pooled for each replicate. Samples from two independent replicates, each containing 10 µg of DNA, were immunoprecipitated with 375 ng (10 µl) of an antibody specific for TCF7L2 (C48H11; 2569, Cell Signaling Technology) or 5 μg of normal rabbit IgG (12-370, Sigma-Aldrich). Incubation with protein G agarose beads and elution of precipitated chromatin was conducted according to ChIP-IT High Sensitivity kit protocol (53040, Activ Motif). Cross-links were reversed and both eluates and input controls were treated with RNAse and Proteinase K, as previously described (Cotney and Noonan, 2015). DNA from two biological replicates was purified with Monarch PCR and DNA Cleanup Kit (T1030, New England Biolabs). Libraries were prepared with KAPA HyperPrep Kit and KAPA Dual-Indexed adapters (7962363001, KK8722, Kapa Biosciences). Enrichment by 15 cycles of amplification was applied and the final library was size-selected using Kapa Pure Beads to obtain the average size of 350 bp. Libraries were tested for quality with the High Sensitivity DNA kit (5067-4626, Agilent) and then were sequenced on an Illumina NovaSeq 6000 instrument in pair-end mode: 2×100 cycles. After trimming, raw reads were mapped to the reference genome, mm10 (UCSC) with the use of BWA (Li and Durbin, 2009). Duplicated reads were identified and removed with the use of the Picard tool (http://broadinstitute.github.io/picard). Peak calling was performed with the use of MACS2 (Zhang et al., 2008). Peaks were assigned to genes with annotatePeak function from the ChIPseeker package using the UCSC Genome Browser. Because TCF7L2 was enriched in intronic regions, we adjusted the annotation algorithm to prioritise the association of peaks with introns. The EdgeR package (Robinson et al., 2010) was used for statistic differential binding analysis between input and TCF7L2 samples. To identify the most significant peaks we filtered the data for *q*-value≤0.01, which resulted in 14,801 peaks annotated to 6728 unique genes. Finally, we filtered the data FE over the input control ≥10, which resulted with 4625 peaks annotated to 3496 unique genes. Motif enrichment and motif discovery analyses were performed using the MEME suite (http://meme-suite.org) on filtered peaks, *q*-value≤0.01, FE≥10. TCF7L2 motif enrichment in ChIP-seq peak sequences was tested with AME (McLeay and Bailey, 2010) using default options. For *de novo* motif discovery, MEME-ChIP (comprising MEME; Bailey and Elkan, 1994), DREME (Bailey, 2011) and CentriMO (Bailey and Machanick, 2012) was used with the DNA HOCOMOCO Mouse (v11 FULL) database (Kulakovskiy et al., 2013; Ma et al., 2014). Statistical significance was tested with Fisher's exact test, and *P-*values were corrected for multiple testing (*E*-value).

### *In vitro* slice electrophysiology

Brain slices (300 μm thick) from control and *Cck^Cre^:Tcf7l2^fl/fl^* mice (four animals per genotype) of both sexes on P21-23 were prepared using an ‘along-row’ protocol in which the anterior end of the brain was cut along a 45° plane toward the midline (Ying and Goldstein, 2005). Slices were cut, recovered and recorded at 24°C in regular artificial cerebrospinal fluid (ACSF) composed of: 119 mM NaCl, 2.5 mM KCl, 1.3 mM MgSO_4_, 2.5 mM CaCl_2_, 1 mM NaH_2_PO_4_, 26.2 mM NaHCO_3_, 11 mM glucose equilibrated with 95/5% O_2_/CO_2_. Somata of thalamocortical neurons (WT *n*=13-16; *Cck^Cre^:Tcf7l2^fl/fl^ n*=17-20) in the VB were targeted for whole-cell patch-clamp recording with borosilicate glass electrodes (resistance 4-8 MΩ). The internal solution was composed of: 125 mM potassium gluconate, 2 mM KCl, 10 mM HEPES, 0.5 mM EGTA, 4 mM MgATP and 0.3 mM NaGTP (pH 7.25-7.35; 290 mOsm). Patch-clamp recordings were collected with a Multiclamp 700B (Molecular Devices) amplifier and Digidata 1550A digitiser and pClamp10.6 (Molecular Devices). Recordings were sampled and filtered at 10 kHz. Analysis of action potentials was performed in Clampfit 10.6. Intensity to Voltage (I-V) plots were constructed from a series of current steps in 40 pA increments from −200 to 600 pA from a holding potential of −65 mV or at the resting membrane potential (∼−57 mV). Two-tailed Mann–Whitney test was used to test for the difference in resting membrane voltage, membrane capacitance, numbers of action potentials and spiking frequency. Two-tailed unpaired Student's *t*-test was used to test for the difference in series resistance (after confirming the normal distribution of the data).

## Supplementary Material

Supplementary information

Reviewer comments

## References

[DEV190181C1] Anders S., Pyl P. T. and Huber W. (2015). HTSeq--a Python framework to work with high-throughput sequencing data. *Bioinformatics* 31, 166-169. 10.1093/bioinformatics/btu638 25260700 PMC4287950

[DEV190181C2] Bailey T. L. (2011). DREME: motif discovery in transcription factor ChIP-seq data. *Bioinformatics* 27, 1653-1659. 10.1093/bioinformatics/btr261 21543442 PMC3106199

[DEV190181C3] Bailey T. L. and Elkan C. (1994). Fitting a mixture model by expectation maximization to discover motifs in biopolymers. *Proc. Int. Conf. Intell. Syst. Mol. Biol.* 2, 28-36. 7584402

[DEV190181C4] Bailey T. L. and Machanick P. (2012). Inferring direct DNA binding from ChIP-seq. *Nucleic Acids Res.* 40, e128 10.1093/nar/gks433 22610855 PMC3458523

[DEV190181C5] Belle M., Godefroy D., Dominici C., Heitz-Marchaland C., Zelina P., Hellal F., Bradke F. and Chédotal A. (2014). A simple method for 3D analysis of immunolabeled axonal tracts in a transparent nervous system. *Cell Rep.* 9, 1191-1201. 10.1016/j.celrep.2014.10.037 25456121

[DEV190181C6] Bem J., Brożko N., Chakraborty C., Lipiec M. A., Koziński K., Nagalski A., Szewczyk Ł. and Wiśniewska M. B. (2019). Wnt/β-catenin signaling in brain development and mental disorders: keeping TCF7L2 in mind. *FEBS Lett.* 593, 1654-1674. 10.1002/1873-3468.13502 31218672 PMC6772062

[DEV190181C7] Benekareddy M., Stachniak T. J., Bruns A., Knoflach F., von Kienlin M., Künnecke B. and Ghosh A. (2018). Identification of a corticohabenular circuit regulating socially directed behavior. *Biol. Psychiatry* 83, 607-617. 10.1016/j.biopsych.2017.10.032 29336819

[DEV190181C8] Beretta C. A., Dross N., Bankhead P. and Carl M. (2013). The ventral habenulae of zebrafish develop in prosomere 2 dependent on Tcf7l2 function. *Neural Dev.* 8, 19 10.1186/1749-8104-8-19 24067090 PMC3827927

[DEV190181C9] Bibollet-Bahena O., Okafuji T., Hokamp K., Tear G. and Mitchell K. J. (2017). A dual-strategy expression screen for candidate connectivity labels in the developing thalamus. *PLoS ONE* 12, e0177977 10.1371/journal.pone.0177977 28558017 PMC5448750

[DEV190181C10] Braisted J. E., Catalano S. M., Stimac R., Kennedy T. E., Tessier-Lavigne M., Shatz C. J. and O'Leary D. D. M. (2000). Netrin-1 promotes thalamic axon growth and is required for proper development of the thalamocortical projection. *J. Neurosci.* 20, 5792-5801. 10.1523/JNEUROSCI.20-15-05792.2000 10908620 PMC6772525

[DEV190181C11] Browne C. A., Hammack R. and Lucki I. (2018). Dysregulation of the lateral habenula in major depressive disorder. *Front. Synaptic Neurosci.* 10, 46 10.3389/fnsyn.2018.00046 30581384 PMC6292991

[DEV190181C12] Cadigan K. M. and Waterman M. L. (2012). TCF/LEFs and Wnt signaling in the nucleus. *Cold Spring Harb. Perspect. Biol.* 4, a007906 10.1101/cshperspect.a007906 23024173 PMC3536346

[DEV190181C13] Chatterjee M., Li K., Chen L., Maisano X., Guo Q., Gan L. and Li J. Y. H. (2012). Gbx2 regulates thalamocortical axon guidance by modifying the LIM and Robo codes. *Development* 139, 4633-4643. 10.1242/dev.086991 23136391 PMC3509725

[DEV190181C14] Chatterjee M., Guo Q., Weber S., Scholpp S. and Li J. Y. H. (2014). Pax6 regulates the formation of the habenular nuclei by controlling the temporospatial expression of Shh in the diencephalon in vertebrates. *BMC Biol.* 12, 13 10.1186/1741-7007-12-13 24528677 PMC3996077

[DEV190181C15] Chen B., Wang S. S., Hattox A. M., Rayburn H., Nelson S. B. and McConnell S. K. (2008). The Fezf2-Ctip2 genetic pathway regulates the fate choice of subcortical projection neurons in the developing cerebral cortex. *Proc. Natl. Acad. Sci. USA* 105, 11382-11387. 10.1073/pnas.0804918105 18678899 PMC2495013

[DEV190181C16] Chen L., Guo Q. and Li J. Y. H. (2009). Transcription factor Gbx2 acts cell-nonautonomously to regulate the formation of lineage-restriction boundaries of the thalamus. *Development* 136, 1317-1326. 10.1242/dev.030510 19279136 PMC2687463

[DEV190181C17] Chen X., Ye R., Gargus J. J., Blakely R. D., Dobrenis K. and Sze J. Y. (2015). Disruption of transient serotonin accumulation by non-serotonin-producing neurons impairs cortical map development. *Cell Rep.* 10, 346-358. 10.1016/j.celrep.2014.12.033 25600870 PMC4824665

[DEV190181C18] Chiara F., Badaloni A., Croci L., Yeh M. L., Cariboni A., Hoerder-Suabedissen A., Consalez G. G., Eickholt B., Shimogori T., Parnavelas J. G. et al. (2012). Early B-cell factors 2 and 3 (EBF2/3) regulate early migration of Cajal-Retzius cells from the cortical hem. *Dev. Biol.* 365, 277-289. 10.1016/j.ydbio.2012.02.034 22421355 PMC3368273

[DEV190181C19] Cho E. A. and Dressler G. R. (1998). TCF-4 binds β-catenin and is expressed in distinct regions of the embryonic brain and limbs. *Mech. Dev.* 77, 9-18. 10.1016/S0925-4773(98)00131-2 9784592

[DEV190181C20] Cho H.-H., Cargnin F., Kim Y., Lee B., Kwon R. J., Nam H., Shen R., Barnes A. P., Lee J. W., Lee S. et al. (2014). Isl1 directly controls a cholinergic neuronal identity in the developing forebrain and spinal cord by forming cell type-specific complexes. *PLoS Genet.* 10, e1004280 10.1371/journal.pgen.1004280 24763339 PMC3998908

[DEV190181C21] Cotney J. L. and Noonan J. P. (2015). Chromatin immunoprecipitation with fixed animal tissues and preparation for high-throughput sequencing. *Cold Spring Harb. Protoc.* 2015, 191-199. 10.1101/pdb.prot084848 25646502

[DEV190181C22] Donovan L. J., Spencer W. C., Kitt M. M., Eastman B. A., Lobur K. J., Jiao K., Silver J. and Deneris E. S. (2019). Lmx1b is required at multiple stages to build expansive serotonergic axon architectures. *eLife* 8, e48788 10.7554/eLife.48788 31355748 PMC6685705

[DEV190181C23] Dufour A., Seibt J., Passante L., Depaepe V., Ciossek T., Frisén J., Kullander K., Flanagan J. G., Polleux F. and Vanderhaeghen P. (2003). Area specificity and topography of thalamocortical projections are controlled by ephrin/Eph genes. *Neuron* 39, 453-465. 10.1016/S0896-6273(03)00440-9 12895420

[DEV190181C24] Ebisu H., Iwai-Takekoshi L., Fujita-Jimbo E., Momoi T. and Kawasaki H. (2016). Foxp2 regulates identities and projection patterns of thalamic nuclei during development. *Cereb. Cortex* 27, 3648-3659. 10.1093/cercor/bhw187 27384060

[DEV190181C25] Evangelio M., García-Amado M. and Clascá F. (2018). Thalamocortical projection neuron and interneuron numbers in the visual thalamic nuclei of the adult C57BL/6 mouse. *Front. Neuroanat.* 12, 27 10.3389/fnana.2018.00027 29706872 PMC5906714

[DEV190181C26] Farley F. W., Soriano P., Steffen L. S. and Dymecki S. M. (2000). Widespread recombinase expression using FLPeR (flipper) mice. *Genesis* 28, 106-110. 10.1002/1526-968X(200011/12)28:3/4<106::AID-GENE30>3.0.CO;2-T 11105051

[DEV190181C27] Fedtsova N. G. and Turner E. E. (1995). Brn-3.0 expression identifies early post-mitotic CNS neurons and sensory neural precursors. *Mech. Dev.* 53, 291-304. 10.1016/0925-4773(95)00435-1 8645597

[DEV190181C28] Fernandez D. C., Fogerson P. M., Lazzerini Ospri L., Thomsen M. B., Layne R. M., Severin D., Zhan J., Singer J. H., Kirkwood A., Zhao H. et al. (2018). Light affects mood and learning through distinct retina-brain pathways. *Cell* 175, 71-84.e18. 10.1016/j.cell.2018.08.004 30173913 PMC6190605

[DEV190181C29] Ferran J. L., Ayad A., Merchán P., Morales- Delgado N., Sánchez-Arrones L., Alonso A., Sandoval J. E., Bardet S. M., Corral-San-Miguel R., Sánchez-Guardado L. Ó. et al. (2015a). Exploring Brain genoarchitecture by single and double chromogenic In Situ Hybridization (ISH) and Immunohistochemistry (IHC) in whole-mount embryos. In *In Situ Hybridization Methods* (ed. G. Hauptmann), pp. 61-82: Humana Press.

[DEV190181C30] Ferran J. L., Ayad A., Merchán P., Morales- Delgado N., Sánchez-Arrones L., Alonso A., Sandoval J. E., Bardet S. M., Corral-San-Miguel R., Sánchez-Guardado L. Ó. et al. (2015b). Exploring brain genoarchitecture by single and double chromogenic In Situ Hybridization (ISH) and Immunohistochemistry (IHC) on cryostat, paraffin, or floating sections. In *In Situ Hybridization Methods*, (ed. G. Hauptmann) pp. 83-107: Humana Press.

[DEV190181C31] Flames N. and Hobert O. (2009). Gene regulatory logic of dopamine neuron differentiation. *Nature* 458, 885-889. 10.1038/nature07929 19287374 PMC2671564

[DEV190181C32] Fremeau R. T., Troyer M. D., Pahner I., Nygaard G. O., Tran C. H., Reimer R. J., Bellocchio E. E., Fortin D., Storm-Mathisen J. and Edwards R. H. (2001). The expression of vesicular glutamate transporters defines two classes of excitatory synapse. *Neuron* 31, 247-260. 10.1016/S0896-6273(01)00344-0 11502256

[DEV190181C33] Frietze S., Wang R., Yao L., Tak Y. G., Ye Z., Gaddis M., Witt H., Farnham P. J. and Jin V. X. (2012). Cell type-specific binding patterns reveal that TCF7L2 can be tethered to the genome by association with GATA3. *Genome Biol.* 13, R52 10.1186/gb-2012-13-9-r52 22951069 PMC3491396

[DEV190181C34] Geoghegan G., Simcox J., Seldin M. M., Parnell T. J., Stubben C., Just S., Begaye L., Lusis A. J. and Villanueva C. J. (2019). Targeted deletion of Tcf7l2 in adipocytes promotes adipocyte hypertrophy and impaired glucose metabolism. *Mol. Metab.* 24, 44-63. 10.1016/j.molmet.2019.03.003 30948248 PMC6531814

[DEV190181C35] Guo Q. and Li J. Y. H. (2019). Defining developmental diversification of diencephalon neurons through single cell gene expression profiling. *Development* 146, dev174284 10.1242/dev.174284 30872278 PMC6602344

[DEV190181C36] Hendricks T. J., Fyodorov D. V., Wegman L. J., Lelutiu N. B., Pehek E. A., Yamamoto B., Silver J., Weeber E. J., Sweatt J. D. and Deneris E. S. (2003). Pet-1 ETS gene plays a critical role in 5-HT neuron development and is required for normal anxiety-like and aggressive behavior. *Neuron* 37, 233-247. 10.1016/S0896-6273(02)01167-4 12546819

[DEV190181C37] Herzog E., Bellenchi G. C., Gras C., Bernard V., Ravassard P., Bedet C., Gasnier B., Giros B. and El Mestikawy S. (2001). The existence of a second vesicular glutamate transporter specifies subpopulations of glutamatergic neurons. *J. Neurosci.* 21, RC181 10.1523/JNEUROSCI.21-22-j0001.2001 11698619 PMC6762292

[DEV190181C38] Hikosaka O. (2010). The habenula: from stress evasion to value-based decision-making. *Nat. Rev. Neurosci.* 11, 503-513. 10.1038/nrn2866 20559337 PMC3447364

[DEV190181C39] Hikosaka O., Sesack S. R., Lecourtier L. and Shepard P. D. (2008). Habenula: crossroad between the basal ganglia and the limbic system. *J. Neurosci.* 28, 11825-11829. 10.1523/JNEUROSCI.3463-08.2008 19005047 PMC2613689

[DEV190181C40] Hirota Y. and Nakajima K. (2017). Control of neuronal migration and aggregation by reelin signaling in the developing cerebral cortex. *Front. Cell Dev. Biol.* 5, 40 10.3389/fcell.2017.00040 28507985 PMC5410752

[DEV190181C41] Hobert O. (2016). Terminal selectors of neuronal identity. *Curr. Top. Dev. Biol.* 116, 455-475. 10.1016/bs.ctdb.2015.12.007 26970634

[DEV190181C42] Hobert O. and Kratsios P. (2019). Neuronal identity control by terminal selectors in worms, flies, and chordates. *Curr. Opin. Neurobiol.* 56, 97-105. 10.1016/j.conb.2018.12.006 30665084

[DEV190181C43] Hüsken U., Stickney H. L., Gestri G., Bianco I. H., Faro A., Young R. M., Roussigne M., Hawkins T. A., Beretta C. A., Brinkmann I. et al. (2014). Tcf7l2 is required for left-right asymmetric differentiation of habenular neurons. *Curr. Biol.* 24, 2217-2227. 10.1016/j.cub.2014.08.006 25201686 PMC4194317

[DEV190181C44] Jager P., Ye Z., Yu X., Zagoraiou L., Prekop H.-T., Partanen J., Jessell T. M., Wisden W., Brickley S. G. and Delogu A. (2016). Tectal-derived interneurons contribute to phasic and tonic inhibition in the visual thalamus. *Nat. Commun.* 7, 13579 10.1038/ncomms13579 27929058 PMC5155147

[DEV190181C45] Jeong Y., Dolson D. K., Waclaw R. R., Matise M. P., Sussel L., Campbell K., Kaestner K. H. and Epstein D. J. (2011). Spatial and temporal requirements for sonic hedgehog in the regulation of thalamic interneuron identity. *Development* 138, 531-541. 10.1242/dev.058917 21205797 PMC3014638

[DEV190181C46] Kadkhodaei B., Ito T., Joodmardi E., Mattsson B., Rouillard C., Carta M., Muramatsu S.-I., Sumi-Ichinose C., Nomura T., Metzger D. et al. (2009). Nurr1 is required for maintenance of maturing and adult midbrain dopamine neurons. *J. Neurosci.* 29, 15923-15932. 10.1523/JNEUROSCI.3910-09.2009 20016108 PMC6666174

[DEV190181C47] Kasten M. R., Rudy B. and Anderson M. P. (2007). Differential regulation of action potential firing in adult murine thalamocortical neurons by Kv3.2, Kv1, and SK potassium and N-type calcium channels. *J. Physiol.* 584, 565-582. 10.1113/jphysiol.2007.141135 17761775 PMC2277158

[DEV190181C48] Kim D., Song I., Keum S., Lee T., Jeong M.-J., Kim S.-S., McEnery M. W. and Shin H.-S. (2001). Lack of the burst firing of thalamocortical relay neurons and resistance to absence seizures in mice lacking α1G T-type Ca(2+) channels. *Neuron* 31, 35-45. 10.1016/S0896-6273(01)00343-9 11498049

[DEV190181C49] Kim D., Langmead B. and Salzberg S. L. (2015). HISAT: a fast spliced aligner with low memory requirements. *Nat. Methods* 12, 357-360. 10.1038/nmeth.3317 25751142 PMC4655817

[DEV190181C50] Kulakovskiy I. V., Medvedeva Y. A., Schaefer U., Kasianov A. S., Vorontsov I. E., Bajic V. B. and Makeev V. J. (2013). HOCOMOCO: a comprehensive collection of human transcription factor binding sites models. *Nucleic Acids Res.* 41, D195-D202. 10.1093/nar/gks1089 23175603 PMC3531053

[DEV190181C51] Lee M., Yoon J., Song H., Lee B., Lam D. T., Baek K., Clevers H. and Jeong Y. (2017). Tcf7l2 plays crucial roles in forebrain development through regulation of thalamic and habenular neuron identity and connectivity. *Dev. Biol.* 424, 62-76. 10.1016/j.ydbio.2017.02.010 28219675

[DEV190181C52] Lehigh K. M., Leonard C. E., Baranoski J. and Donoghue M. J. (2013). Parcellation of the thalamus into distinct nuclei reflects EphA expression and function. *Gene Expr. Patterns* 13, 454-463. 10.1016/j.gep.2013.08.002 24036135 PMC3839050

[DEV190181C53] Li H. and Durbin R. (2009). Fast and accurate short read alignment with Burrows-Wheeler transform. *Bioinformatics* 25, 1754-1760. 10.1093/bioinformatics/btp324 19451168 PMC2705234

[DEV190181C54] Li K., Zhang J. and Li J. Y. H. (2012). Gbx2 plays an essential but transient role in the formation of thalamic nuclei. *PLoS ONE* 7, e47111 10.1371/journal.pone.0047111 23056596 PMC3464241

[DEV190181C55] Liu C., Maejima T., Wyler S. C., Casadesus G., Herlitze S. and Deneris E. S. (2010). Pet-1 is required across different stages of life to regulate serotonergic function. *Nat. Neurosci.* 13, 1190-1198. 10.1038/nn.2623 20818386 PMC2947586

[DEV190181C56] Lodato S., Molyneaux B. J., Zuccaro E., Goff L. A., Chen H.-H., Yuan W., Meleski A., Takahashi E., Mahony S., Rinn J. L. et al. (2014). Gene co-regulation by Fezf2 selects neurotransmitter identity and connectivity of corticospinal neurons. *Nat. Neurosci.* 17, 1046-1054. 10.1038/nn.3757 24997765 PMC4188416

[DEV190181C57] Love M. I., Huber W. and Anders S. (2014). Moderated estimation of fold change and dispersion for RNA-seq data with DESeq2. *Genome Biol.* 15, 550 10.1186/s13059-014-0550-8 25516281 PMC4302049

[DEV190181C58] López-Bendito G. (2018). Development of the thalamocortical interactions: past, present and future. *Neuroscience* 385, 67-74. 10.1016/j.neuroscience.2018.06.020 29932982 PMC7611009

[DEV190181C59] Ma W., Noble W. S. and Bailey T. L. (2014). Motif-based analysis of large nucleotide data sets using MEME-ChIP. *Nat. Protoc.* 9, 1428-1450. 10.1038/nprot.2014.083 24853928 PMC4175909

[DEV190181C60] Mallika C., Guo Q. and Li J. Y. H. (2015). Gbx2 is essential for maintaining thalamic neuron identity and repressing habenular characters in the developing thalamus. *Dev. Biol.* 407, 26-39. 10.1016/j.ydbio.2015.08.010 26297811 PMC4641819

[DEV190181C61] McLeay R. C. and Bailey T. L. (2010). Motif Enrichment Analysis: a unified framework and an evaluation on ChIP data. *BMC Bioinformatics* 11, 165 10.1186/1471-2105-11-165 20356413 PMC2868005

[DEV190181C62] Miyashita-Lin E. M., Hevner R., Wassarman K. M., Martinez S. and Rubenstein J. L. (1999). Early neocortical regionalization in the absence of thalamic innervation. *Science* 285, 906-909. 10.1126/science.285.5429.906 10436162

[DEV190181C63] Nagalski A., Irimia M., Szewczyk L., Ferran J. L., Misztal K., Kuznicki J. and Wisniewska M. B. (2013). Postnatal isoform switch and protein localization of LEF1 and TCF7L2 transcription factors in cortical, thalamic, and mesencephalic regions of the adult mouse brain. *Brain Struct. Funct.* 218, 1531-1549. 10.1007/s00429-012-0474-6 23152144 PMC3825142

[DEV190181C64] Nagalski A., Puelles L., Dabrowski M., Wegierski T., Kuznicki J. and Wisniewska M. B. (2016). Molecular anatomy of the thalamic complex and the underlying transcription factors. *Brain Struct. Funct.* 221, 2493-2510. 10.1007/s00429-015-1052-5 25963709 PMC4884203

[DEV190181C65] Nakagawa Y. (2019). Development of the thalamus: from early patterning to regulation of cortical functions. *Wiley Interdiscip. Rev. Dev. Biol.* 275, e345 10.1002/wdev.345 31034163

[DEV190181C66] Nishimura S., Bilgüvar K., Ishigame K., Sestan N., Günel M. and Louvi A. (2015). Functional synergy between cholecystokinin receptors CCKAR and CCKBR in mammalian brain development. *PLoS ONE* 10, e0124295 10.1371/journal.pone.0124295 25875176 PMC4398320

[DEV190181C67] Norton L., Fourcaudot M., Abdul-Ghani M. A., Winnier D., Mehta F. F., Jenkinson C. P. and Defronzo R. A. (2011). Chromatin occupancy of transcription factor 7-like 2 (TCF7L2) and its role in hepatic glucose metabolism. *Diabetologia* 54, 3132-3142. 10.1007/s00125-011-2289-z 21901280

[DEV190181C69] Pereira L., Kratsios P., Serrano-Saiz E., Sheftel H., Mayo A. E., Hall D. H., White J. G., LeBoeuf B., Garcia L. R., Alon U. et al. (2015). A cellular and regulatory map of the cholinergic nervous system of C. elegans. *eLife* 4, e12432 10.7554/eLife.12432 26705699 PMC4769160

[DEV190181C70] Phillips J. W., Schulmann A., Hara E., Winnubst J., Liu C., Valakh V., Wang L., Shields B. C., Korff W., Chandrashekar J. et al. (2019). A repeated molecular architecture across thalamic pathways. *Nat. Neurosci.* 22, 1925-1935. 10.1038/s41593-019-0483-3 31527803 PMC6819258

[DEV190181C71] Puelles L., Morales-Delgado N., Merchán P., Castro-Robles B., Martínez-de-la-Torre M., Díaz C. and Ferran J. L. (2016). Radial and tangential migration of telencephalic somatostatin neurons originated from the mouse diagonal area. *Brain Struct. Funct.* 221, 3027-3065. 10.1007/s00429-015-1086-8 26189100 PMC4920861

[DEV190181C72] Puelles L., Diaz C., Stühmer T., Ferran J. L., Martínez-de la Torre M. and Rubenstein J. L. R. (2020). LacZ-reporter mapping of Dlx5/6 expression and genoarchitectural analysis of the postnatal mouse prethalamus. *J. Comp. Neurol.* 2020, 1-54. 10.1002/cne.24952 PMC7671952 32420617

[DEV190181C73] Quina L. A., Wang S., Ng L. and Turner E. E. (2009). Brn3a and Nurr1 mediate a gene regulatory pathway for habenula development. *J. Neurosci.* 29, 14309-14322. 10.1523/JNEUROSCI.2430-09.2009 19906978 PMC2802832

[DEV190181C74] Robinson M. D., McCarthy D. J. and Smyth G. K. (2010). edgeR: a Bioconductor package for differential expression analysis of digital gene expression data. *Bioinformatics* 26, 139-140. 10.1093/bioinformatics/btp616 19910308 PMC2796818

[DEV190181C75] Serrano-Saiz E., Poole R. J., Felton T., Zhang F., De La Cruz E. D. and Hobert O. (2013). Modular control of glutamatergic neuronal identity in C. elegans by distinct homeodomain proteins. *Cell* 155, 659-673. 10.1016/j.cell.2013.09.052 24243022 PMC3855022

[DEV190181C76] Serrano-Saiz E., Leyva-Díaz E., De La Cruz E. and Hobert O. (2018). BRN3-type POU homeobox genes maintain the identity of mature postmitotic neurons in nematodes and mice. *Curr. Biol.* 28, 2813-2823.e2812. 10.1016/j.cub.2018.06.045 30146154

[DEV190181C77] Sherman S. M. (2017). Functioning of circuits connecting thalamus and cortex. *Compr. Physiol.* 7, 713-739. 10.1002/cphy.c160032 28333385

[DEV190181C78] Shi W., Xianyu A., Han Z., Tang X., Li Z., Zhong H., Mao T., Huang K. and Shi S.-H. (2017). Ontogenetic establishment of order-specific nuclear organization in the mammalian thalamus. *Nat. Neurosci.* 20, 516-528. 10.1038/nn.4519 28250409 PMC5374008

[DEV190181C79] Shimogori T., Lee D. A., Miranda-Angulo A., Yang Y., Wang H., Jiang L., Yoshida A. C., Kataoka A., Mashiko H., Avetisyan M. et al. (2010). A genomic atlas of mouse hypothalamic development. *Nat. Neurosci.* 13, 767-775. 10.1038/nn.2545 20436479 PMC4067769

[DEV190181C80] Skarnes W. C., Rosen B., West A. P., Koutsourakis M., Bushell W., Iyer V., Mujica A. O., Thomas M., Harrow J., Cox T. et al. (2011). A conditional knockout resource for the genome-wide study of mouse gene function. *Nature* 474, 337-342. 10.1038/nature10163 21677750 PMC3572410

[DEV190181C81] Steullet P. (2019). Thalamus-related anomalies as candidate mechanism-based biomarkers for psychosis. *Schizophr. Res.* (in press) 10.1016/j.schres.2019.05.027 31147286

[DEV190181C82] Taniguchi H., He M., Wu P., Kim S., Paik R., Sugino K., Kvitsiani D., Kvitsani D., Fu Y., Lu J. et al. (2011). A resource of Cre driver lines for genetic targeting of GABAergic neurons in cerebral cortex. *Neuron* 71, 995-1013. 10.1016/j.neuron.2011.07.026 21943598 PMC3779648

[DEV190181C83] Tapia M., Baudot P., Formisano-Tréziny C., Dufour M. A., Temporal S., Lasserre M., Marquèze-Pouey B., Gabert J., Kobayashi K. and Goaillard J.-M. (2018). Neurotransmitter identity and electrophysiological phenotype are genetically coupled in midbrain dopaminergic neurons. *Sci. Rep.* 8, 13637 10.1038/s41598-018-31765-z 30206240 PMC6134142

[DEV190181C84] Tran H. N., Park W., Seong S., Jeong J. E., Nguyen Q. H., Yoon J., Baek K. and Jeong Y. (2020). Tcf7l2 transcription factor is required for the maintenance, but not the initial specification, of the neurotransmitter identity in the caudal thalamus. *Dev. Dyn.* 249, 646-655. 10.1002/dvdy.146 31872525

[DEV190181C85] Vacik T., Stubbs J. L. and Lemke G. (2011). A novel mechanism for the transcriptional regulation of Wnt signaling in development. *Genes Dev.* 25, 1783-1795. 10.1101/gad.17227011 21856776 PMC3175715

[DEV190181C86] Vitalis T., Dauphinot L., Gressens P., Potier M.-C., Mariani J. and Gaspar P. (2017). RORα coordinates thalamic and cortical maturation to instruct barrel cortex development. *Cereb. Cortex* 28, 3994-4007. 10.1093/cercor/bhx262 29040410

[DEV190181C87] Walther C. and Gruss P. (1991). Pax-6, a murine paired box gene, is expressed in the developing CNS. *Development* 113, 1435-1449. 1687460 10.1242/dev.113.4.1435

[DEV190181C88] Watson C., Paxinos G. and Puelles L. (2012). *The Mouse Nervous System*. Academic Press.

[DEV190181C89] Whiting B. B., Whiting A. C. and Whiting D. M. (2018). Thalamic deep brain stimulation. *Prog. Neurol. Surg.* 33, 198-206. 10.1159/000481104 29332084

[DEV190181C90] Wisniewska M. B., Misztal K., Michowski W., Szczot M., Purta E., Lesniak W., Klejman M. E., Dabrowski M., Filipkowski R. K., Nagalski A. et al. (2010). LEF1/β-catenin complex regulates transcription of the Cav3.1 calcium channel gene (Cacna1g) in thalamic neurons of the adult brain. *J. Neurosci.* 30, 4957-4969. 10.1523/JNEUROSCI.1425-09.2010 20371816 PMC6632775

[DEV190181C91] Wisniewska M. B., Nagalski A., Dabrowski M., Misztal K. and Kuznicki J. (2012). Novel β-catenin target genes identified in thalamic neurons encode modulators of neuronal excitability. *BMC Genomics* 13, 635 10.1186/1471-2164-13-635 23157480 PMC3532193

[DEV190181C92] Wong S. Z. H., Scott E. P., Mu W., Guo X., Borgenheimer E., Freeman M., Ming G.-L., Wu Q.-F., Song H. and Nakagawa Y. (2018). In vivo clonal analysis reveals spatiotemporal regulation of thalamic nucleogenesis. *PLoS Biol.* 16, e2005211 10.1371/journal.pbio.2005211 29684005 PMC5933804

[DEV190181C93] Woodward N. D., Giraldo-Chica M., Rogers B. and Cascio C. J. (2017). Thalamocortical dysconnectivity in autism spectrum disorder: an analysis of the Autism Brain Imaging Data Exchange. *Biol. Psychiatry Cogn. Neurosci. Neuroimaging* 2, 76-84. 10.1016/j.bpsc.2016.09.002 28584881 PMC5455796

[DEV190181C94] Wyler S. C., Spencer W. C., Green N. H., Rood B. D., Crawford L. T., Craige C., Gresch P., McMahon D. G., Beck S. G. and Deneris E. (2016). Pet-1 switches transcriptional targets postnatally to regulate maturation of serotonin neuron excitability. *J. Neurosci.* 36, 1758-1774. 10.1523/JNEUROSCI.3798-15.2016 26843655 PMC4737783

[DEV190181C95] Ying S.-W. and Goldstein P. A. (2005). Propofol suppresses synaptic responsiveness of somatosensory relay neurons to excitatory input by potentiating GABA_A_ receptor chloride channels. *Mol. Pain* 1, 2 10.1186/1744-8069-1-2 15813991 PMC1074352

[DEV190181C96] Yuge K., Kataoka A., Yoshida A. C., Itoh D., Aggarwal M., Mori S., Blackshaw S. and Shimogori T. (2011). Region-specific gene expression in early postnatal mouse thalamus. *J. Comp. Neurol.* 519, 544-561. 10.1002/cne.22532 21192083

[DEV190181C97] Zhang Y., Liu T., Meyer C. A., Eeckhoute J., Johnson D. S., Bernstein B. E., Nussbaum C., Myers R. M., Brown M., Li W. et al. (2008). Model-based analysis of ChIP-Seq (MACS). *Genome Biol.* 9, R137 10.1186/gb-2008-9-9-r137 18798982 PMC2592715

[DEV190181C98] Zhang F., Bhattacharya A., Nelson J. C., Abe N., Gordon P., Lloret-Fernandez C., Maicas M., Flames N., Mann R. S., Colón-Ramos D. A. et al. (2014). The LIM and POU homeobox genes ttx-3 and unc-86 act as terminal selectors in distinct cholinergic and serotonergic neuron types. *Development* 141, 422-435. 10.1242/dev.099721 24353061 PMC3879818

