# Supplementary material for: TCF7L2 regulates postmitotic differentiation programmes and excitability patterns in the thalamus
Source: Development. 2020 Aug 25;147(16):dev190181. doi: 10.1242/dev.190181 (PMC7473649; doi:10.1242/dev.190181)
Supplement: Supplementary information [file develop-147-190181-s1.pdf]

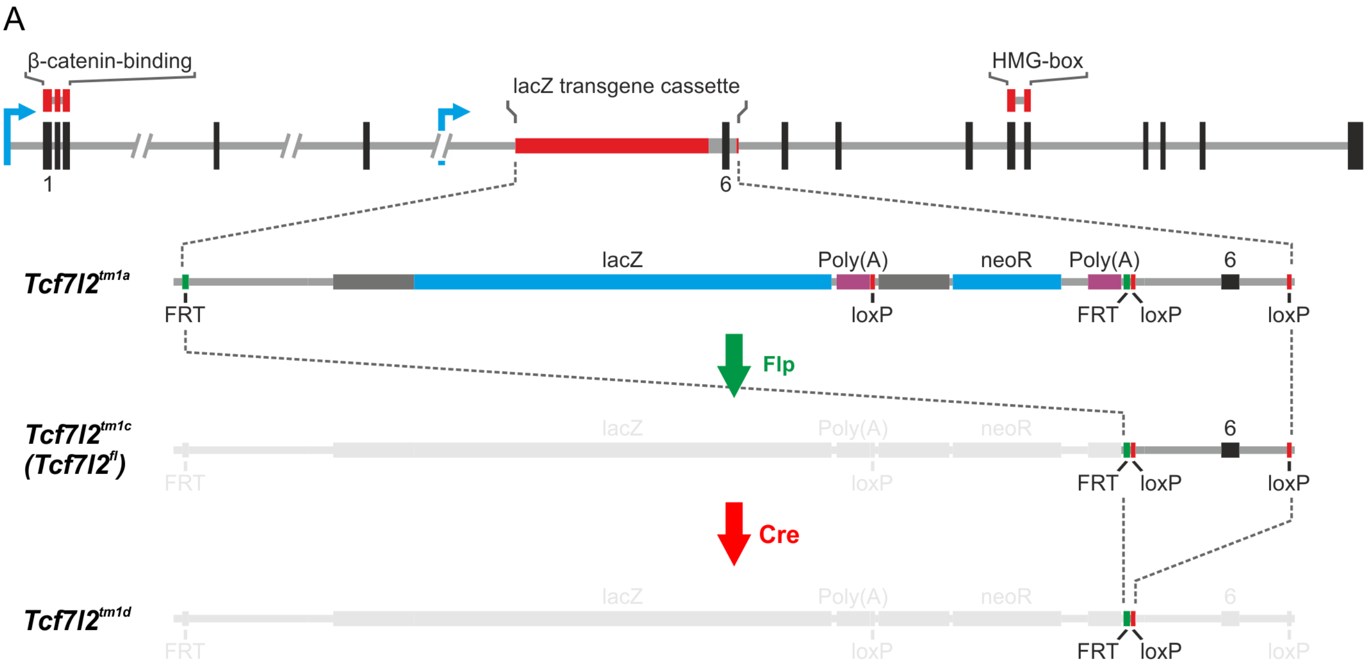

Figure S1. Schematic representation of *Tcf7l2*^{tm1a} allele and its conversion into *Tcf7l2*^{tm1d} allele. *Tcf7l2*^{tm1a} allele has a trap cassette with the *lacZ* and *neoR* elements inserted upstream of the critical exon 6 of the *Tcf7l2* gene. Expression from the *Tcf7l2*^{tm1a} leads to the production of a truncated *Tcf7l2* mRNA and lack of functional TCF7L2 protein. Mice homozygous for the *Tcf7l2*^{tm1a} allele were used as *Tcf7l2*$^{-/-}$ knockout mice. After crossing this strain with mice expressing Flippase, the *lacZ* and *neoR* elements were removed from the *Tcf7l2*^{tm1a} allele, thus creating *Tcf7l2*^{tm1c} allele with its critical exon 6 flanked with loxP sites. Mice homozygous for the *Tcf7l2*^{tm1c} allele (*Tcf7l2*$^{fl/fl}$) were then crossed with a strain expressing Cre recombinase driven by the Cholecystokinin gene promoter (*Cck*^{Cre}). The expression of *Cck* and *Tcf7l2* overlaps in the thalamus. In the resulting *Cck*^{Cre}*:Tcf7l2*$^{fl/fl}$ strain, *Tcf7l2* is knocked out in *Cck*-positive thalamic neurons. Exons and introns are represented by vertical black and horizontal grey lines, respectively. Blue arrows indicate transcription start sites. Regions that encode the β-catenin binding domain and HMG-box are marked by red lines above the exons.

Development • Supplementary information

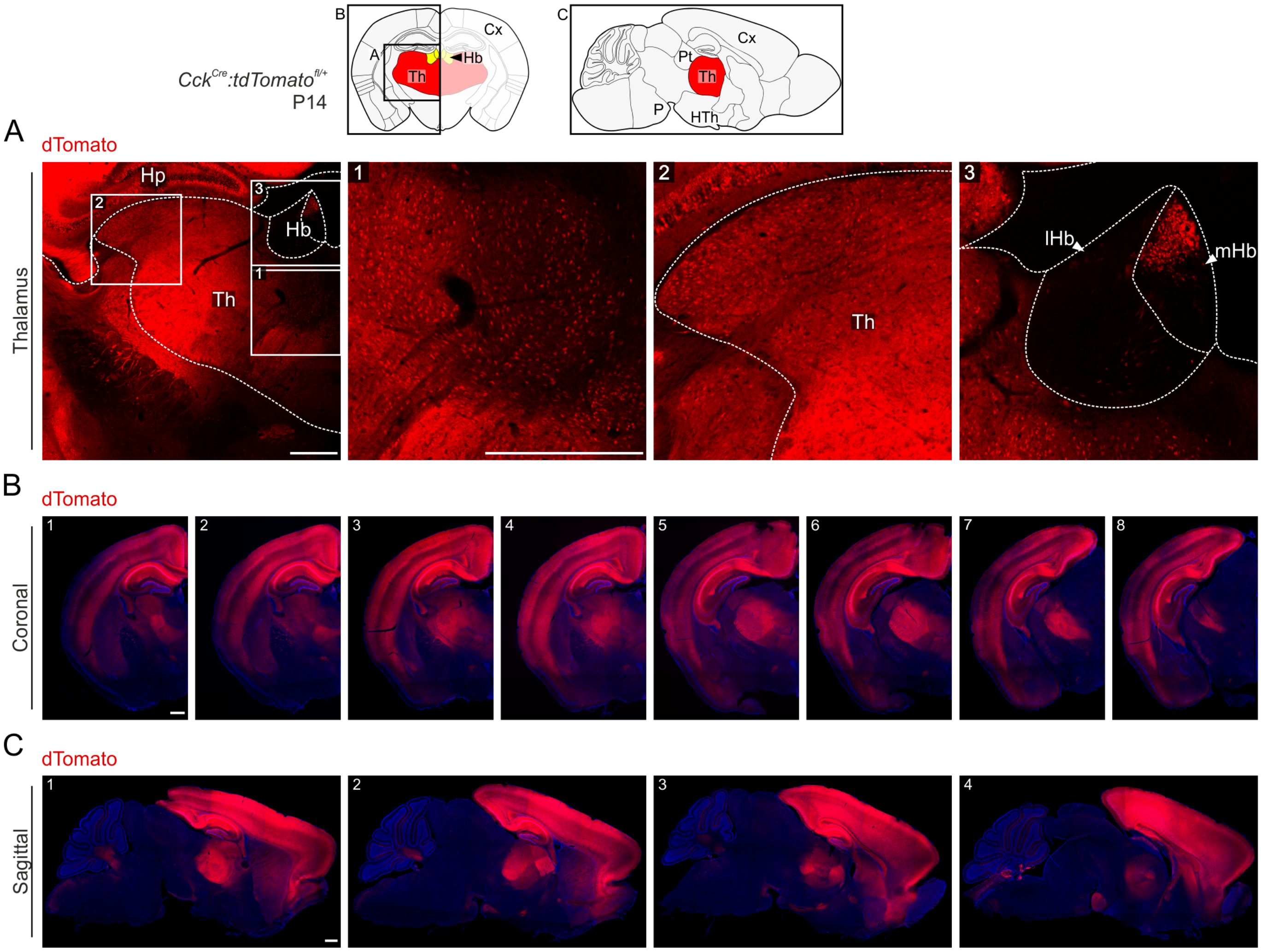

Figure S2. *Cck^Cre* expression in the mouse brain on P14. Expression of tdTomato in *Cck^Cre:tdTomato^fl/+* reporter line in **(A)** a single thalamic section, and in consecutive **(B)** coronal and **(C)** sagittal brain sections. Cx, cortex; Hb, habenula; Hp, hippocampus; HTh, hypothalamus; lHb, lateral habenula; mHb, medial habenula; P, pons; Pt, pretectum; Th, thalamus. Scale bars represent 0.5 mm.

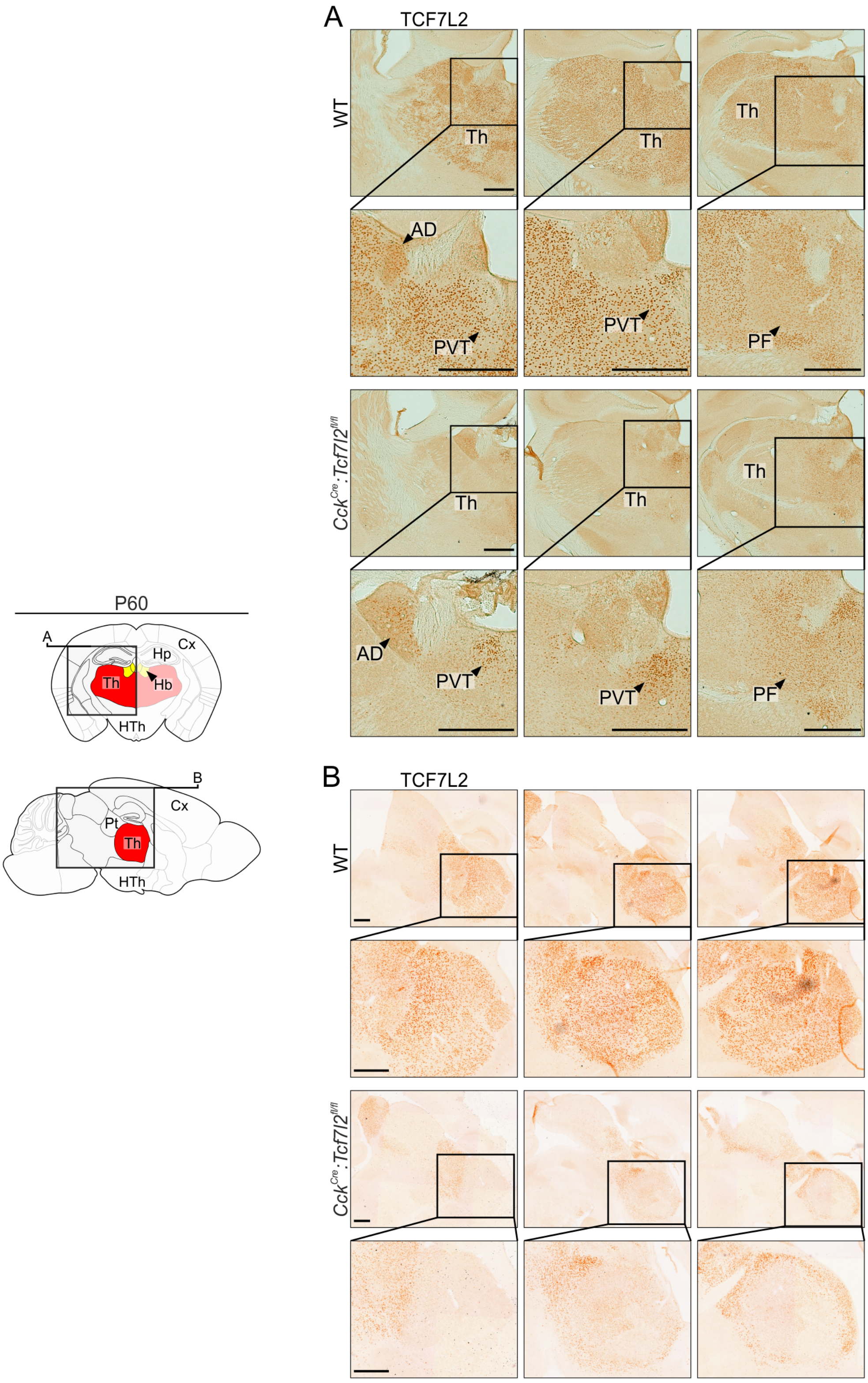

Figure S3. Depletion of the TCF7L2 protein in the thalamus of *Cck*^{Cre}:*Tcf7l2*$^{fl/fl}$ on P60. DAB immunohistochemical staining of TCF7L2 in consecutive **(A)** coronal and **(B)** sagittal brain sections from WT and *Cck*^{Cre}:*Tcf7l2*$^{fl/fl}$ mice. TCF7L2 is successfully depleted in majority of the majority of thalamic nuclei The magnified views show TCF7L2-positive cells in the anterodorsal nucleus (AD), paraventricular nucleus (PVT) and parafascicular nucleus (PF). Cx, cortex; Hb, habenula; Hp, hippocampus; HTh, hypothalamus; lHb, lateral habenula; mHb, medial habenula; Pt, pretectum; Th, thalamus. Scale bars represent 0.5 mm.

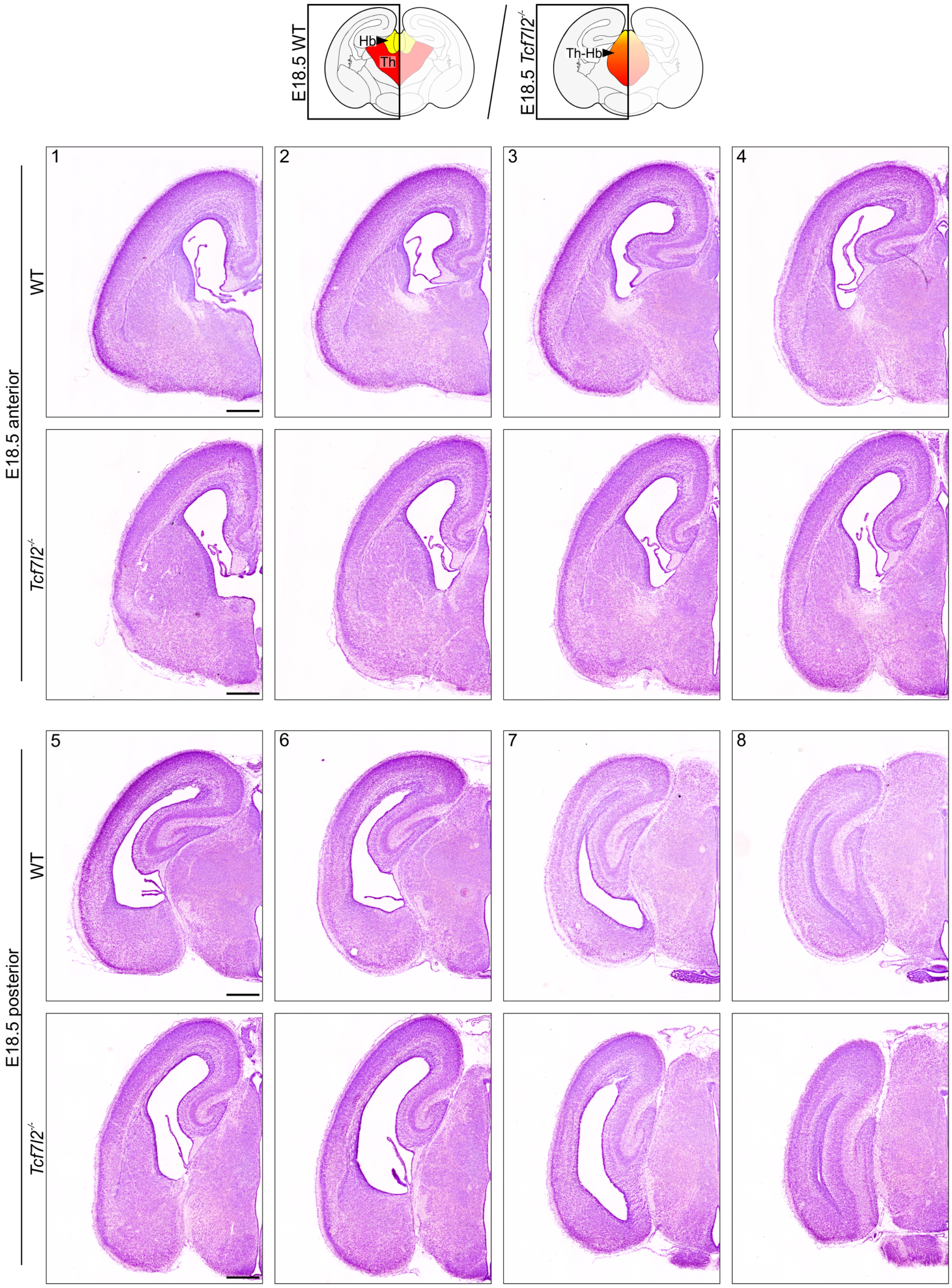

Figure S4. Anatomy of the E18.5 *Tcf7l2*$^{-/-}$ brain. Nissl staining in consecutive coronal brain sections of E18.5 WT and *Tcf7l2*$^{-/-}$ embryos on E18.5. Hb, habenula; Th, thalamus; Th-Hb, thalmo-habenular region. Scale bars represent 0.5 mm.

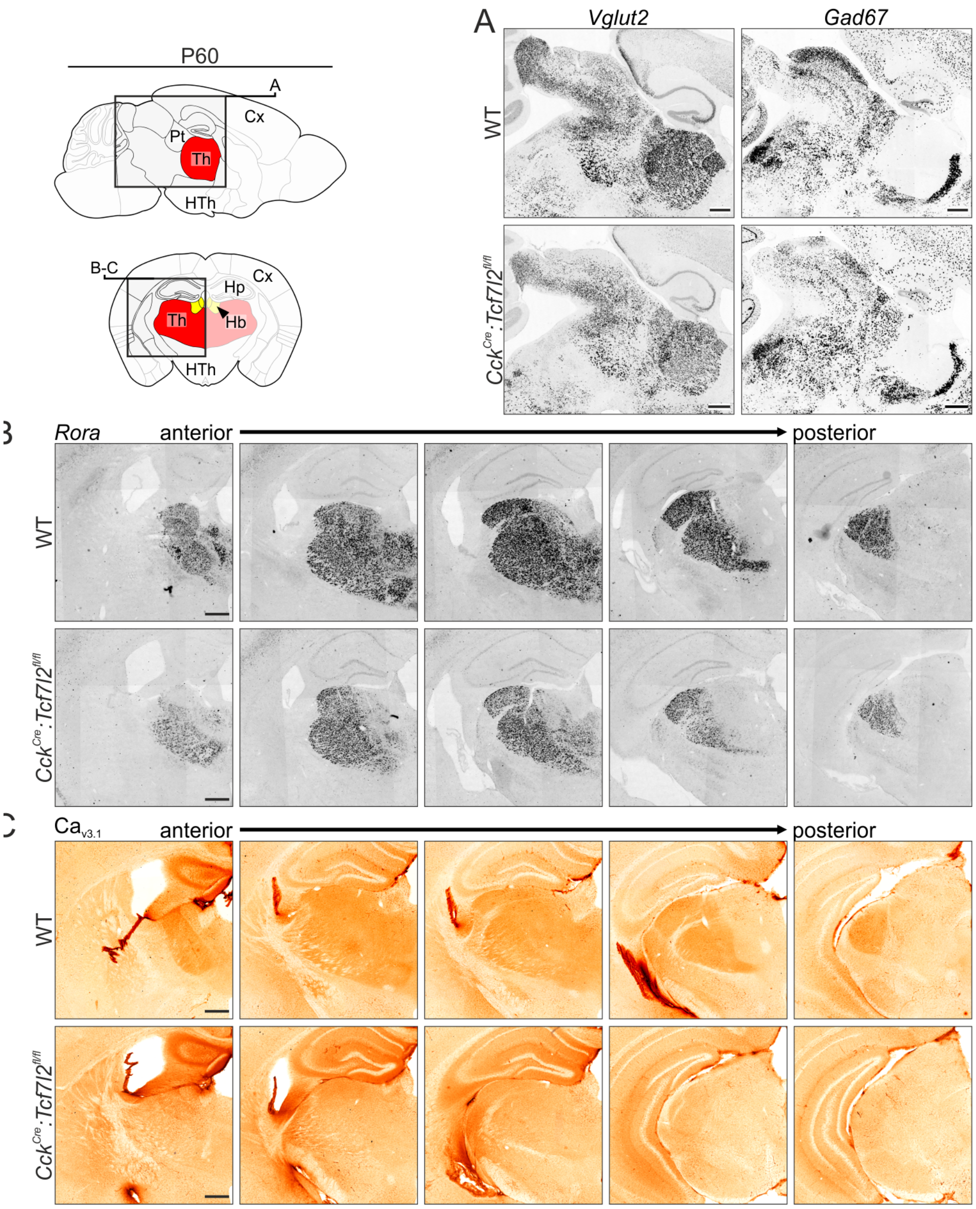

Figure S5. Expression of the Ca$_{v3.1}$ protein and *Vglut2, Gad67,* and *Rora* mRNA in P60 *Cck^{Cre}:Tcf7l2$^{fl/fl}$*. (A) *In situ* hybridisation with *Vglut2/Slc17a6* and *Gad1/Gad67* probes in sagittal brain sections. **(B)** *In situ* hybridisation with *Rora* probe in consecutive coronal brain sections. **(C)** DAB immunohistochemical staining of Ca$_{v3.1}$ in consecutive coronal brain sections. Cx, cortex; Hb, habenula; Hp, hippocampus; HTh, hypothalamus; Pt, pretectum; Th, thalamus. Scale bars represent 0.5 mm.

Development • Supplementary information

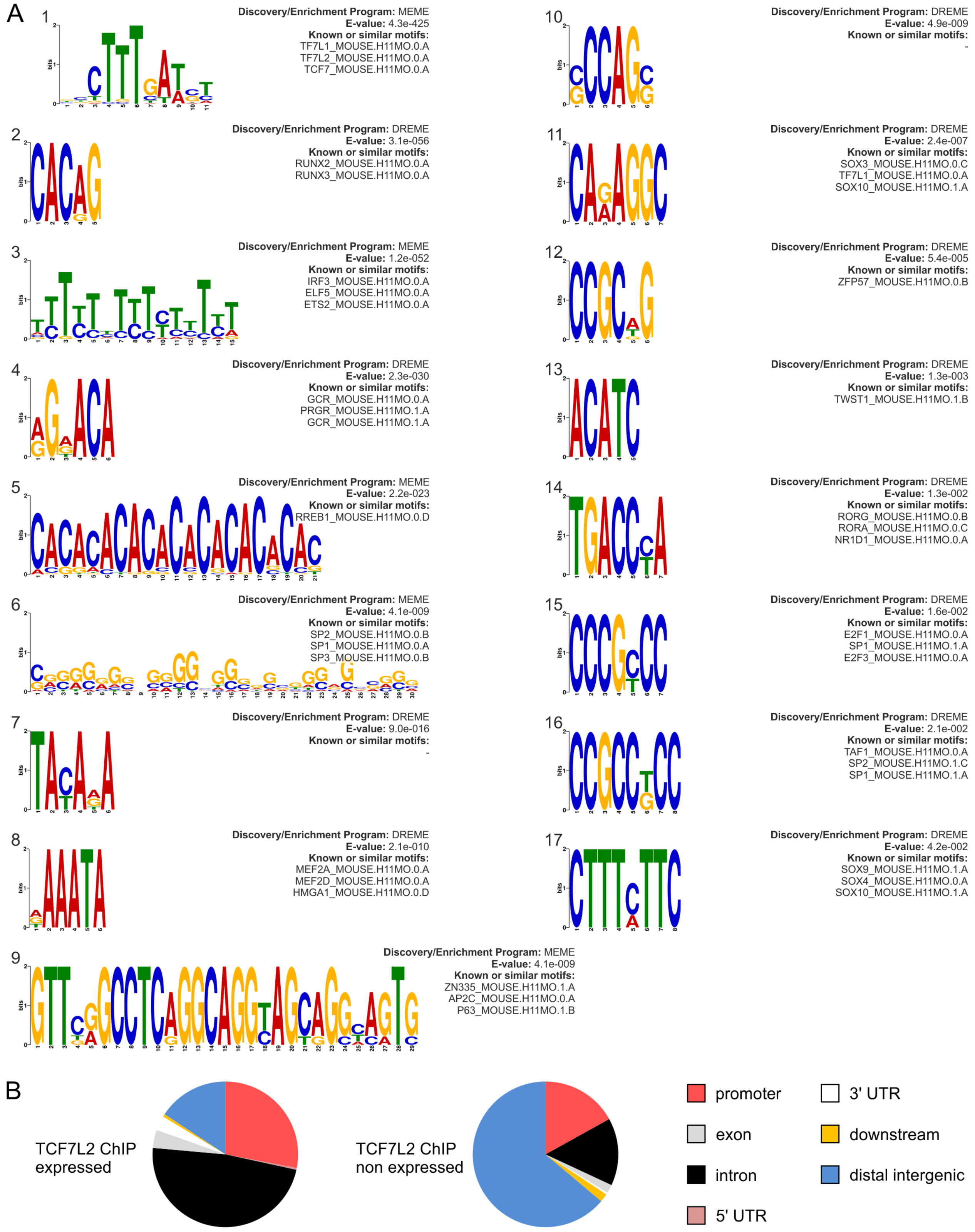

Figure S6. Motif discovery and genomic distribution analysis of TCF7L2 ChIP peaks. (A) *De novo* motif discovery in 4624 TCF7L2 ChIP-seq peaks with MEME-ChIP. Logos of significant *de novo* motifs, *E* values, and similarities to motifs from HOCOMOCO database (mouse) are shown. **(B)** Genomic distribution of the TCF7L2 binding sites. Genes that were and were not expressed on P60 were analysed separatedly.

Development • Supplementary information

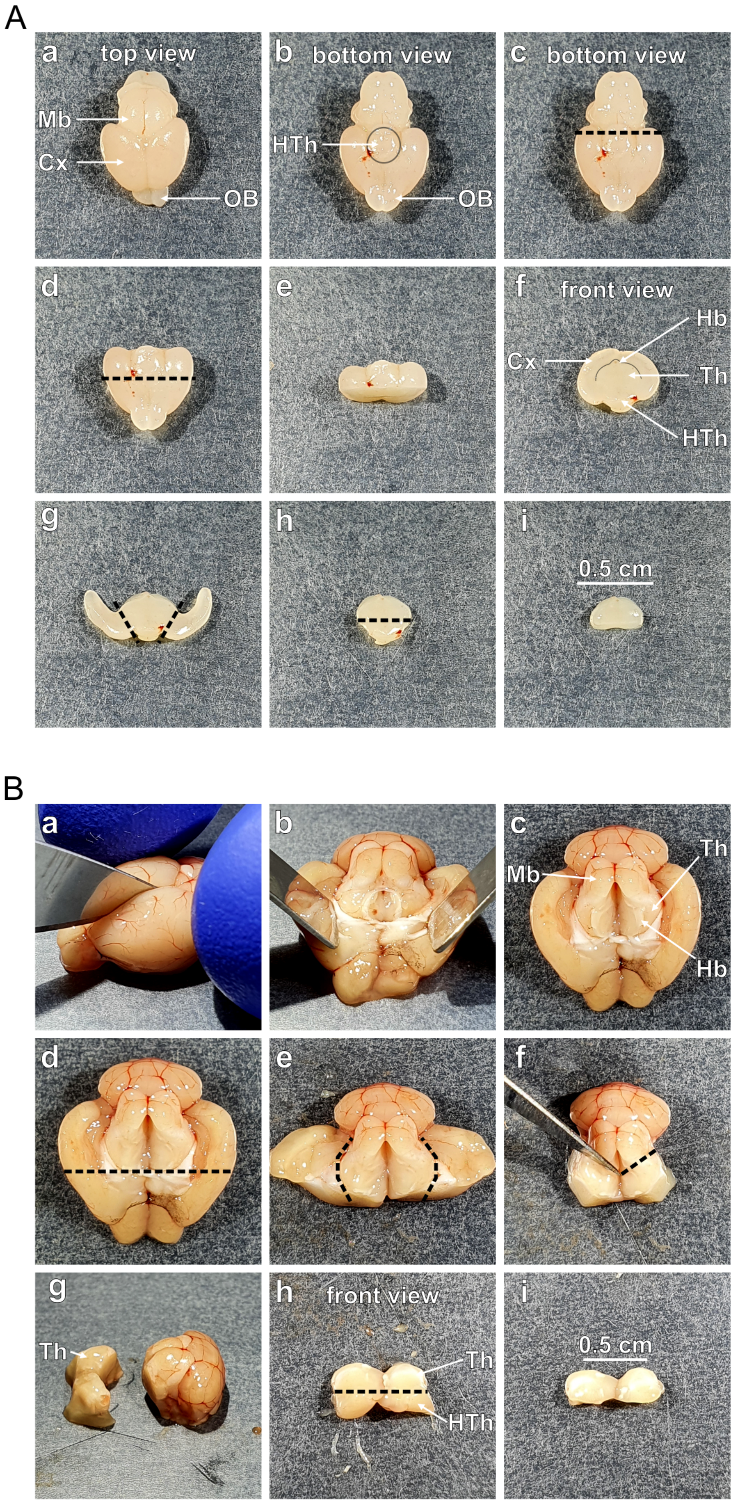

Development • Supplementary information

Figure S7. Sectioning of the thalamo-habenular region. (A) Sectioning of the thalamo-habenular region from the embryonic brain (E18.5). (a-e) A brain slice containing the thalamus and habenula is dissected by cutting the brain from the bottom view through the preoptic area close to the optic chiasm and through the mammillary bodies; (f-g) The pallium is pulled apart with a spatula and cut away; (h-i) The hypothalamus is removed with a straight cut below the thalamus. **(B)** Sectioning of the thalamo-habenular region from the adult brain (P60). (a-c) The cortical hemispheres are cut open and pulled apart with a spatula to expose the subcortical structures including thalamus and habenula; (d-e) The pallium and subpallium are removed with cuts at the front and on the sides of the thalamus; (f) The midbrain and hindbrain are removed with a V-shaped cut at the back of the thalamus, made at the level of the pretectum; (g-h) The hypothalamus is removed with a straight cut below the thalamus. Cx, cortex; Hb, habenula; HTh, hypothalamus; Mb, midbrain; OB, olfactory bulb; Th, thalamus.

Table S1. RNA-seq data for E18.5 wild type and *Tcf7l2*$^{-/-}$ mice (DeSeq2). all_expressed_WT_KO_E18.5 - lists of all genes with non 0 normalised values or non NA *p* values; E18.5_sign._log2FC<-0.4_or_>0.4 - differentially expressed genes (*q* value ≤ 0.05) with arbitrary $\log_2$ fold change cutoffs ≥ 0.4 or ≤ -0.4. n=3 (independent biological replicates).

Click here to Download Table S1

Table S2. Gene ontology enrichment analysis of the E18.5 DEGs. Reported GO terms (*q* value ≤ 0.01) are divided into 3 ontologies: biological process, molecular function and cellular component.

Click here to Download Table S2

Table S3. RNA-seq data for P60 wild type and *Cck*^{Cre}:*Tcf7l2*$^{fl/fl}$ mice (DeSeq2). all_expressed_WT_KO_E18.5_P60 - lists of all genes with non 0 normalised values or non NA *p* values ion E18.5 or P60; all_expressed_WT_KO_P60 - list of all differentially expressed genes on P60; P60_sign._log2FC<-0.4_or_>0.4 - differentially expressed genes (*q* value ≤ 0.05) with arbitrary $\log_2$ fold change cutoffs ≥ 0.4 or ≤ -0.4. n=3 (independent biological replicates).

Click here to Download Table S3

Development • Supplementary information

Table S4. Gene ontology enrichment analysis of the P60 DEGs. Reported GO terms (*q* value ≤ 0.01) are divided into 3 ontologies: biological process, molecular function and cellular component.

Click here to Download Table S4

Table S5. TCF7L2 ChIP-Seq data for P60 wild type mice. 14801 significant (*q* value ≤ 0,01), annotated to 6728 unique genes; 4624 peaks with arbitrary fold enrichement ≥ 10, intersected with the P60 DEGs from the RNA-seq analysis. Two independent biological replicates, for each replicates; chromatin from 6 mice was pooled for each replicate.

Click here to Download Table S5

Table S6. Gene ontology enrichment analysis of the genes identified by the TCFL2 ChIP-seq and expressed in the thalamo-habenular region in wild type mice on P60. Reported GO terms (*q* value ≤ 0.01) are divided into 3 ontologies: biological process, molecular function and cellular component.

Click here to Download Table S6
