# Supplementary material for: TCF7L2 regulates postmitotic differentiation programmes and excitability patterns in the thalamus
Source: Development. 2020 Aug 25;147(16):dev190181. doi: 10.1242/dev.190181 (PMC7473649; doi:10.1242/dev.190181)
Supplement: Reviewer comments [file develop-147-190181_review_history.pdf]

TCF7L2 regulates postmitotic differentiation programmes and excitability patterns in the thalamus

Marcin Andrzej Lipiec, Joanna Bem, Kamil Koziński, Chaitali Chakraborty, Joanna Urban-Ciećko, Tomasz Zajkowski, Michał Dąbrowski, Łukasz Mateusz Szewczyk, Angel Toval, José Luis Ferran, Andrzej Nagalski and Marta Barbara Wiśniewska
DOI: 10.1242/dev.190181

Editor: Francois Guillemot

Review timeline
Original submission: 4 March 2020
Editorial decision: 9 April 2020
First revision received: 8 July 2020
Accepted: 8 July 2020

Original submission

First decision letter

MS ID#: DEVELOP/2020/190181

MS TITLE: TCF7L2 regulates postmitotic differentiation programs and excitability patterns in the thalamus

AUTHORS: Marcin Andrzej Lipiec, Kamil Kozinski, Tomasz Zajkowski, Joanna Bem, Joanna Urban-Ciecko, Michal Dabrowski, Chaitali Chakraborty, Lukasz Mateusz Szewczyk, Angel Toval, Jose Luis Ferran, Andrzej Nagalski, and Marta Barbara Wisniewska

I have now received the reports of three referees on your manuscript and I have reached a decision. The referees' comments are appended below, or you can access them online: please go to BenchPress and click on the 'Manuscripts with Decisions' queue in the Author Area.

As you will see, all the referees are enthusiastic about your work, but they also have some criticisms and recommend a substantial revision of your manuscript before we can consider publication. If you are able to revise the manuscript along the lines suggested, which may involve further experiments, I will be happy to receive a revised version of the manuscript. Your revised paper may be re-reviewed by the original referees, and its acceptance will depend on your addressing satisfactorily all their major concerns. Please also note that Development will normally permit only one round of major revision.

We are aware that you may currently be unable to access the lab to undertake experimental revisions. If it would be helpful, we encourage you to contact us to discuss your revision in greater detail. Please send us a point-by-point response indicating where you are able to address concerns raised (either experimentally or by changes to the text) and where you will not be able to do so within the normal timeframe of a revision. We will then provide further guidance. Please also note that we are happy to extend revision timeframes as necessary.

Please attend to all of the reviewers' comments and ensure that you clearly highlight all changes made in the revised manuscript. Please avoid using 'Tracked changes' in Word files as these are lost in PDF conversion. I should be grateful if you would also provide a point-by-point response detailing

© 2020. Published by The Company of Biologists under the terms of the Creative Commons Attribution License (https://creativecommons.org/licenses/by/4.0/).

how you have dealt with the points raised by the reviewers in the 'Response to Reviewers' box. If you do not agree with any of their criticisms or suggestions please explain clearly why this is so.

Reviewer 1

Advance summary and potential significance to field

TCF7L2 is a member of the LEF1/TCF transcription factor family (Cadigan & Waterman, 2012) expressed in prosomere 2 neurons (Nagalski et al., 2013, 2016). Its expression is maintained throughout life and TCF7L2 motifs are overrepresented in putative enhancers of adult thalamus-enriched genes (Nagalski et al., 2016; Wisniewska et al., 2012). Based on these data, Lipiec et al. in the present manuscript propose that this factor can be a prosomere 2 terminal selector gene.

The work by Lipiec et al. describes rigorously the role of TCF7L2 in neuronal differentiation of thalamic glutamatergic neurons at two developmental stages, highlighting its involvement in the postnatal establishment of critical thalamic electrophysiological features.

A previous paper by Lee et al in 2017 already showed that TCF7L2 does not influence the proliferation and neurogenesis of thalamic and habenula neurons but influences the connectivity in the thalamo-habenular region which is disrupted in TCF7L2 KO embryos. The present manuscript confirms those results by using a different knock-out model. The authors show that cell clustering in the thalamo-habenular region is altered when TCF7L2 is absent, giving rise to the lack of boundaries between prosomere 2 and neighboring structures. In addition, they analyzed the transcriptome of the prosomere 2 in E18.5 knock-out mice and found significant changes in the expression of genes involved in the regulation of transcription factor (TF) activity, anatomical structure development, neuronal differentiation, axon guidance, cell adhesion, regulation of cell migration, and synaptic signaling. Importantly, the expression of TF such as Rora, Lef1, Foxp2, and Prox1 which are subregional thalamic markers was virtually absent in the KO animals. Moreover, the expression of habenular markers Lef1 and Etv1 was abolished.

In order to investigate the function of TCF7L2 at the thalamus during postnatal and adult stages, the authors generated a thalamic conditional TCF7L2 KO mice in which the downregulation of TCF7L2 starts at P4. They characterized the GABAergic and glutamatergic cell distribution in this model at P60 (and also in the full KO at E18.5) and found no alterations, meaning that TCF7L2 is not involved in the specification and maintenance of VGLUT2-identity in prosomere 2.

To investigate whether TCF7L2 regulates terminal gene batteries, they performed an RNA-seq of the thalamus of the cKO at P60 and found significant changes in the expression of genes related to synaptic proteins and regulators of membrane conductance. In addition, by ChIP-seq they showed that many of these genes are direct targets of TCF7L2. As 90% of these genes are induced after embryogenesis, these results support the function of TCF7L2 as a terminal selector during postnatal development.

Finally, the authors demonstrate that TCF7L2 expression is physiologically relevant. By performing patch-clamp recordings in acute slices they showed alterations in the input resistance, evoke action potentials and rebound bursts of postnatal thalamic neurons in the cKO mice. The authors conclude that TCF7L2 is essential for the establishment of unique excitability and firing patterns in thalamocortical neurons.

Overall, the manuscript is rigorous, well-executed and complete. The conclusions made by the author are well drawn by their experimental data and the manuscript deserves publication.

Comments for the author

Overall, the manuscript is rigorous, well-executed and complete. The conclusions made by the author are well drawn by their experimental data and the manuscript deserves publication.

1) The authors focus on the role of TCF7L2 in prosomere 2. However, this gene is also expressed in p1

© 2020. Published by The Company of Biologists under the terms of the Creative Commons Attribution License (https://creativecommons.org/licenses/by/4.0/).

(pretectum), p3 (prethalamus) and midbrain (colliculus and istmus), even at postnatal stages. Can the authors explain if the phenotype they observed can be in part due to the function of this gene at these other structures?

2) This study shows that the induction of genes that confer electrophysiological features of thalamic neurons is impaired in postnatal TCF7L2 KO. By ChIP-Seq the authors showed that a 38% of the upregulated and 22% of the downregulated genes are direct targets of TCF7L2, however, could the authors speculate about the possible mechanism driving the differential expression of the additional altered genes? Are these genes part of a same cascade? Are those changes a consequence of the change in the expression of additional TFs?

3) Can the authors specify how do they precisely dissect the prosomere 2 at E18.5 for their transcriptomic experiments?

4) The authors performed RNA-Seq on the prosomere 2 of the TCF7L2 KO by E18.5. Although that´s not the main goal of the paper, I would suggest them to analyze the transcriptome of the KO at earlier stages in order to confirm that this TF is not involved in the proliferation and/or neurogenesis of that region.

5) Why the authors perform the transcriptomics experiments at P60-P75 while the electrophysiology is done at P21-P23?

6) A probe for TCF7L2 is used to identify prosomere 2 in WT and KO embryos by in situ hybridization. Is it RNA expression of TCF7L2 preserved in KO animals? Is that probe directed against a region of the gene conserved in the KO? Please clarify this point.

7) The number of mice used per experiment should be identified. From how many litters, how many animals are contained in a sample in the case of immunohistochemistry, Nissl, in situ hybridization, DiI and Western Blot; In the case of the RNA-Seq and ChIP-Seq the authors mention that they used RNA/DNA samples from 3/2 independent biological replicates, does it mean from 3 animals from 3 different litters? from individual mice? from a pool of individuals?

- Additional minor comments and typos.

-The authors clearly explain in the text that, by using the Cck-Cre;Tcf7l2-Flox mice, the expression of TCF7L2 starts to be downregulated in the thalamus by P4 and the removal of the protein is complete by P14. In the schema in Figure 2G, arrows from P4 and P14 point to "Complete TCF7L2 removal" which is confusing. I would suggest to modify this point to make it clearer.

-Typo: change "ani-PAX6" by "anti-PAX6 in page 7 "Impaired cell clustering in the diencephalon" section.

-Typo: change "performer" by "performed" in the 4th line of page 21.

Reviewer 2

Advance summary and potential significance to field

In this paper, the authors characterize the function of the transcription factor TCF7L2 in thalamic and habenular neurons through a comprehensive analysis in newly generated full and conditional Tcf7l2 knockout mice. Mutant analysis indicated that TCF7L2 is required for proper neuroanatomy and axonal connectivity in the thalamic and habenular regions.
Transcriptional profiling in Tcf7l2 knockout mice revealed the deregulation of thalamic and habenular regulatory networks and several effector genes. The transcriptome of thalamic conditional mutants (complete depletion in the thalamus by P14) additionally showed impaired expression of excitability and synaptic genes induced postnatally. Through genome-wide binding profiling, the authors demonstrated that TCF7L2 binds directly to terminal genes in the thalamus. Finally, using electrophysiological recordings in slices they showed that TCF7L2 is required for normal firing patterns in thalamic neurons.

© 2020. Published by The Company of Biologists under the terms of the Creative Commons Attribution License (https://creativecommons.org/licenses/by/4.0/).

The approaches taken by the authors are rigorous and deep and they use a number of advanced approaches to document the role of TCF7L2 as a master regulator of the thalamic region. The authors need to be commended for this very rigorous analysis. This reviewer has no major concerns or questions the quality of the work presented here, but has noticed some minor points listed below.

Comments for the author

1) In Figure 3 the authors noted a reduction in the radial dimension of the whole thalamic region, which is quite apparent in the images shown in this figure. However, in Figure 3B no significant changes in proliferation were observed through KI-67 quantification, if anything even a tendency for more KI-67-positive cells in the mutants. How do the authors reconcile these two observations? Are these cells generated and then die?

2) Fig. 2B. In the WT image, TCF7L2 expression in the habenula is unclear in this image (e.g. compare with expression in 2G or 1B). Is this a matter of the rostrocaudal level of the section? The authors should consider using a more representative image.

3) When discussing the Tcf7l2tm1a allele in the main text, the authors mentioned “ectopic expression of β-galactosidase from the lacZ locus”. The authors should consider showing this expression in Figure 2.

4) Fig. 3C. This is unclear, are these consecutive coronal sections? In addition, it seems that the thalamus is invading habenular territory, but not the opposite.

5) Fig. 3E. Labelled as P0 in the figure panel but E18.5 in the legend and main text.

6) Fig. 3K. Mutant images are of higher magnification than WT images. For proper comparison, lower magnification mutant images should be shown here. There is space to actually show both, a low-magnification image of the entire area, and higher magnification pictures for the habenula for both WT and mutant at 2 different anterio-posterior levels.

7) Main text about TCF7L2 direct binding: “suggesting that TCF7L2 acts directly as an activator rather than repressor”. TCF7L2 direct binding to upregulated genes also suggests direct role in repression. Without further evidence this suggestion should be revised.

<u>Reviewer 3</u>

Advance summary and potential significance to field

In this manuscript Lipiec and collaborators investigate the role of the transcription factor Tcf2l7 in the specification of a general pan-thalamic neuron identity, using constitutive and conditional inactivation of Tcf2l7 in combination with in situ marker detection, tissue RNA sequencing, chromatin immunoprecipitation and whole cell patch clamp recordings. In part, their analysis extends to the epithalamus and its habenular derivatives, however these are not investigated as extensively and comprehensively as the thalamic (dorsal thalamus) region.
The findings can be divided into two areas: one relating to embryonic development and one dealing with the postnatal acquisition of definitive thalamic functional properties. While the embryonic function of Tcf7l2 especially in the context of thalamic-epithalamic identity and tract formation (thalamo-cortical and fasciculus retroflexus), was the subject of previous publications (see in particular Lee et al. Dev Bio 2017), this manuscript adds important novel information on the transcriptional changes that take place in the thalamic-epithalamic territory -intended as the derivative of the second prosomere- upon constitutive Tcf7l2 inactivation. The transcriptional changes observed upon postnatal inactivation of Tcf7l2 are, to my knowledge, entirely novel and will benefit future research in the field of late thalamic development and maturation.

© 2020. Published by The Company of Biologists under the terms of the Creative Commons Attribution License (https://creativecommons.org/licenses/by/4.0/).

Comments for the author

The following conceptual elements should be discussed in a revised manuscript:

-The authors interpret their findings as to indicate that Tcf7l2 is a terminal selector gene for all thalamic neurons. This conclusion impinges mostly on analysis of prospective thalamo-cortical neurons and certainly the defects in electrical properties in glutamatergic neurons of the ventrobasal complex at P21 support this interpretation. However, Tcf7l2 is also expressed in the derivatives of the rTh domain of the second prosomere and these are fundamentally different cells in terms of neurotransmitter/neuropeptide expression cell membrane properties and long-range axonal connectivity. Interestingly, several of the genes upregulated upon Tcf7l2 deletion at E18.5 are expressed in the rTh derivatives (e.g. Nkx2.2, Lhx5, Reelin) possibly highlighting an altogether different role of Tcf7l2 in this subdomain of prosomere 2. The authors should discuss whether they consider Tcf7l2 as required to provide pan-thalamic neuronal features that are shared among excitatory and inhibitory neurons alike, referring to the relevant experiment evidence.

-Again, on the subject of how generalisable the findings are, the authors should discuss, and if additional evidence is available include it in the manuscript, whether the prospective midline thalamus should be included in their generalisation that Tcf7l2 is a terminal selector for the entire thalamus. Based on Cck RNA in situ data available on the Allen Brain Atlas, the Cck driver may not be active in certain thalamo-cortical nuclei especially along the midline (i.e. PVN, reuniens, centromedial).

-Expression levels of Tcf7l2 are clearly different in certain areas: the authors acknowledge this for the rTh derivatives IGL and vLGN, but similar considerations should also apply to prospective thalamo-cortical nuclei as can be seen for instance in Figure 1B WT panel, Figure 2B WT panel and Figure 2G WT P14 panel. The authors should articulate their “results” and “discussion” sections of the paper in view of potential differential requirements for Tcf7l2 in different thalamo-cortical nuclei.

The data are generally compelling and clearly presented, with some exceptions that require addressing and that are listed below:

-Images of thalamic tissue in the coronal plane are meant to illustrate an anterior and a posterior level. However, examples are fairly anterior and, in some cases, only one anterior section is provided. Figures 2G Figure 6A (P60) and 6C, 6E should be revised to include examples of more caudal levels (i.e. at level of medial geniculate, posterior and full extension of the lateral geniculate complex).

-The authors should comment on the relationship between the prethalamic cells visible at E12.5 in Figure 1A and the vLGN. Can the authors confidently say that all the Tcf7l2 positive cells in the vLGN are prosomere 2 derived?

-Can the authors comment on the Tcf7l2 negative area lateral to the habenular complex visible clearly in Figure 1B and its inset n.1? The area corresponds to the recently described perihabenula (Fernandez et al. Cell 2018)- a novel nucleus of the thalamus. Could this nucleus depend on a distinctive differentiation program?

-Optional- Would the authors be in the position to add data to Figure 2 illustrating Cck-cre activity, using a suitable reporter line? This is not essential but would help refining the overall interpretation of the gene expression data.

-Figure 3C. There appears to be an increase in the number of Pou4F1 cells, not just a spread into thalamic territory. The authors should comment on this finding and also address whether they think there is a conversion of thalamic neurons into epithalamic ones or an intermingling of thalamic and epithalamic neurons.

-Figure 3F (but also 4B) seems to suggest that the obvious migratory defect in the prethalamic Pax6 population (lateral thalamus/prethalamus) is cell-extrinsic. Can the authors comment further on that?

© 2020. Published by The Company of Biologists under the terms of the Creative Commons Attribution License (https://creativecommons.org/licenses/by/4.0/).

-Figure 3J,K: similar data already published by Lee et al. Dev Bio 2017. I am not convinced that panels J and K should be included.

-Figure 5. The authors should measure enrichment for rTh and cTH specific genes in the downregulated and upregulated cluster. Based on a scan-through the gene list, it would appear that while epithalamic and cTh genes are downregulated in the Tcf7l2 knockout, rTh are upregulated. If so, how can this be interpreted?

-Figure 7B. The Venn diagram should be modified to include all ChIP enriched genes, not just those that are contained within the transcriptionally regulated groups.

-Discuss putative Tcf7l2 transcriptional targets identified here in relation to transcriptional targets previously identified in Lee et al. Dev Bio 2017 and LEF1/TCF targets previously identified by the senior author particularly Cacna1G Wisniewska et al. J Neurosci 2010.

Methodological considerations (mandatory revision in my view):

-There is insufficient data on the quality of the material used for the ChIP experiment. How specific is the TCf7l2 antibody used? The authors should show biochemical validation of the IP and include a negative control, for instance the TCf7l2 knockout thalamus sample, where the IP shouldn't pull down any DNA.

-Figure 5 A. there are inconsistencies with the first sample out of the three knockout replicates. Does this reflect biological variation or the dissection method? The description of the dissection method at E18.5 and P60 should be provided in greater detail. Where there any post-dissection controls carried out to assess prosomere 2 specificity of the samples used?

First revision

<u>Author response to reviewers' comments</u>

Reviewer 1 Comments for the authorand our answers:

1) The authors focus on the role of TCF7L2 in prosomere 2. However, this gene is also expressed in p1 (pretectum), p3 (prethalamus) and midbrain (colliculus and istmus), even at postnatal stages. Can the authors explain if the phenotype they observed can be in part due to the function of this gene at these other structures?

Results on CckCre:Tcf7l2fl/fl mice demonstrate specific consequences of Tcf7l2 loss in the thalamic region postnatally, because the expression of Tcf7l2 and Cck overlaps only in the thalamus (and the rostral part of the medial habenula). However, it is true that the effects of Tcf7l2 knockout on other brain structures cannot be discarded in embryos with the full knockout. We commented it as follow:

Page 14 (Discussion)
Presumably, cell non-autonomous and secondary mechanisms contribute to morphological malformation of the thalamo-habenular region. (…) Considering that topographic axonal connections can create physical boundaries in the developing brain, disorganised stria medullaris or afferent connections from the retina, pretectum and midbrain, where Tcf7l2 is expressed at high levels, may also play a role.

2) This study shows that the induction of genes that confer electrophysiological features of thalamic neurons is impaired in postnatal TCF7L2 KO. By ChIP-Seq the authors showed that a 38% of the upregulated and 22% of the downregulated genes are direct targets of TCF7L2, however, could the authors speculate about the possible mechanism driving the differential expression of the

© 2020. Published by The Company of Biologists under the terms of the Creative Commons Attribution License (https://creativecommons.org/licenses/by/4.0/).

additional altered genes? Are these genes part of a same cascade? Are those changes a consequence of the change in the expression of additional TFs?

Some genes in this group have statistically significant ChIP-seq peaks, but these peaks did not meet our threshold parameters. However, the majority were not annotated to the identified peaks. To explore possible mechanisms driving differential expression of these genes, we performed motif enrichment analysis in their putative regulatory elements (mouse-to-human conserved sequences +/- 10kb from transcription start sites). The TCF7L2 motif was overrepresented in the DEG/ChIP-seq group (q-value 0.002) but not in DEG/nonChIP-seq group, confirming that the latter are not likely to include many direct targets of TCF7L2 or that they are regulated by distal enhancers with TCF7L2 binding sites. Secondary transcription factors may also play a role in the regulation of TCF7L2-target genes (direct and indirect) Indeed, the DEGs with annotated ChIP-seq peaks are enriched with motifs of several thalamic transcription factors, in addition to the TCF7L2 motif. We added this explanation and new result in the current version of the manuscript, which reads as follow:

Page 11 (Results)

The remaining peaks (i.e., annotated to non-expressed genes) were mainly annotated to predicted genes and pseudogenes, and were located in distal intergenic regions, suggesting that they represent distal enhancers of unidentified genes.
(...)
The DEGs with no annotated ChIP-seq peaks can be indirect targets of TCF7L2 or are regulated by TCF7L2-dependent distal enhancers.
(...)
The motifs of GCR (NR3C1), RREB1 and RORA were also overrepresented (E value = 1.3-32, 2.3-30, 2.2-23, respectively). These transcription factors are enriched in the thalamus, their expression was altered in CckCre:Tcf7l2fl/fl mice, and their genes were identified by the ChIP-seq, suggesting that not only are they downstream targets of TCF7L2 but also cooperates with TCF7L2 in gene expression regulation.

Page 15 (Discussion)
Cooperation with subregional thalamic transcription factors, such as RORA, NR3C1 and RREB1, as suggested by the overrepresentation of the corresponding binding motifs in the TCF7L2 ChIP-seq peaks, could contribute to TCF7L2-dependent regulation of differentially expression thalamic genes, but this question needs further investigation.

3) Can the authors specify how do they precisely dissect the prosomere 2 at E18.5 for their transcriptomic experiments?

We illustrated and explained the dissection procedure in supplemental figures:

Sectioning of the talamo-habenular region from the embryonic brain (E18.5)
A-E) A brain slice containing the thalamus and habenula is dissected by cutting the brain from the bottom view through the preoptic area close to the optic chiasm and through the mammillary bodies;
F-G) The pallium is pulled apart with a spatula and cut away;
H-I) The hypothalamus is removed with diagonal and straight cuts at the front of and below the thalamus. For knockout thalami, a lower section of similar size is cut away;
Sectioning of the thalamo-habenular region from the adult brain
A-C) The pallial hemispheres and corpus callosum are cut and pulled apart with a spatula to expose subcortical structures;
D-E) The pallium and striatum are removed with cuts at the front and on the sides of the thalamus;
F) The midbrain and hindbrain are removed with a V-shaped cut at the back of the thalamus, made at the level of the pretectum;
K-L) The hypothalamus is removed with straight cuts below the thalamus.

4) The authors performed RNA-Seq on the prosomere 2 of the TCF7L2 KO by E18.5. Although that´s not the main goal of the paper, I would suggest them to analyse the transcriptome of the KO at

© 2020. Published by The Company of Biologists under the terms of the Creative Commons Attribution License (https://creativecommons.org/licenses/by/4.0/).

earlier stages in order to confirm that this TF is not involved in the proliferation and/or neurogenesis of that region.

We agree that it would be interesting to add RNA-seq data for Tcf7l2 KO mice at different embryonic stages (in addition to E18.5 and P60). However, earlier stages of thalamic development were a focus of study by Lee et al. 2017. These authors did not observe proliferation/neurogenesis impairments or alterations in the expression of Shh and progenitor markers Pax6, Neurog1, Neurog2 Olig2, Olig3, Ascl2, Dlx2, Dlx5, Helt, Gata2 and Tal2 in the brain on E12.5. We hope that the reviewer will agree that RNA-seq on E12.5 is not essential in our work and is not likely to provide critical information.

5) Why the authors perform the transcriptomics experiments at P60-P75 while the electrophysiology is done at P21-P23?

We performed ChIP-seq on P60 to analyse TCF7L2-chromatin interactions in the fully mature thalamus. RNA-seq was performed at the same age to match ChIP-seq. Electrophysiology, in turn, was performed on P21-23, because preparation of healthy acute slices and clamping neurons is much more consistent in the younger brain than fully myelinated adult brain. This analysis was possible because thalamic “excitability/neurotransmission” genes, such as Cacna1g, Kcnc2 and Gabra4, are already expressed at high levels in WT brains at this stage, and the CckCre-driven Tcf7l2 knockout in the mutant animals is completed at least 7 days prior to the study.

6) A probe for TCF7L2 is used to identify prosomere 2 in WT and KO embryos by in situ hybridization. Is it RNA expression of TCF7L2 preserved in KO animals? Is that probe directed against a region of the gene conserved in the KO? Please clarify this point.

We clarified it as follow:

Page 19 (Materials and Methods)
The Tcf7l2 probe spans the first 8 exons of Tcf7l2 gene, therefore it detects also Tcf7l2 transcripts which are truncated after exon 5 in the mutant mice.

7) The number of mice used per experiment should be identified. From how many litters, how many animals are contained in a sample in the case of immunohistochemistry, Nissl, in situ hybridization, DiI and Western Blot; In the case of the RNA-Seq and ChIP-Seq the authors mention that they used RNA/DNA samples from 3/2 independent biological replicates, does it mean from 3 animals from 3 different litters? from individual mice? from a pool of individuals?

This important information was indeed missing, and now is included in method descriptions and figure legends.

Page 19 (Materials and Methods)
3-5 embryos/mice per genotype from at least two litters were used in each analysis by Nissl staining, DiI axon tracing, immunohistochemistry or in situ hybridisation.

Page 20 (Materials and Methods)
Protein extracts were obtained from 6 animals per genotype from at least two litters.

Page 21 (Materials and Methods)
RNA samples from three animals (two litters) for each genotype were sequenced (...)

Page 21 (Materials and Methods)
Chromatin from 6 mice (two litters) was pooled for each replicate. (...)

Additional minor comments and typos.
-The authors clearly explain in the text that, by using the Cck-Cre;Tcf7l2-Flox mice, the expression of TCF7L2 starts to be downregulated in the thalamus by P4 and the removal of the protein is complete by P14. In the schema in Figure 2G, arrows from P4 and P14 point to “Complete TCF7L2 removal” which is confusing. I would suggest to modify this point to make it clearer.

© 2020. Published by The Company of Biologists under the terms of the Creative Commons Attribution License (https://creativecommons.org/licenses/by/4.0/).

-Typo: change “ani-PAX6” by “anti-PAX6 in page 7 “Impaired cell clustering in the diencephalon” section.
-Typo: change “performer” by “performed” in the 4th line of page 21.

Thank you, this was corrected.

Reviewer 2 Comments for the authorand our answers:

1) In Figure 3 the authors noted a reduction in the radial dimension of the whole thalamic region, which is quite apparent in the images shown in this figure. However, in Figure 3B, no significant changes in proliferation were observed through KI-67 quantification, if anything even a tendency for more KI-67-positive cells in the mutants. How do the authors reconcile these two observations? Are these cells generated and then die?

We do not think that thalamic/habenular neurons die in Tcf7l2-/- embryos. Firstly, Lee et al. (2017) showed that the number of cleaved caspase 3-positive cells in prosomere 2 was similar in WT and Tcf7l2-/- embryos on E12.5. Secondly, apoptosis-related genes were not overrepresented in the group of genes that were differentially expressed in the thalamo-habenular region on E18.5 in our RNA-seq analysis. We think that the thalamohabenular area was not decreased, because it was elongated along the dorsoventral axis. We included a series of Nissl stained sections in supplements to support this conclusion, and described as follow:

Results (page 6)
The whole region was reduced in the radial dimension and elongated dorsoventrally, resulting in an oval-like shape.
2) Fig. 2B. In the WT image, TCF7L2 expression in the habenula is unclear in this image (e.g. compare with expression in 2G or 1B). Is this a matter of the rostrocaudal level of the section? The authors should consider using a more representative image.
We agree and replaced the image (Fig. 2B).

3) When discussing the Tcf7l2tm1a allele in the main text, the authors mentioned “ectopic expression of β-galactosidase from the lacZ locus”. The authors should consider showing this expression in Figure 2.

We agree with this comment and modified the Figure 2 accordingly.

4) Fig. 3C. This is unclear, are these consecutive coronal sections? In addition, it seems that the thalamus is invading habenular territory, but not the opposite.

Yes, these are consecutive sections of the same brain. We added this information in the figure description. We also replaced the section in Fig. 3C with a more representative one. In the series of sections (below), it is apparent that Gbx2+ and POU4F1+ areas extend into each other.

5) Fig. 3E. Labelled as P0 in the figure panel but E18.5 in the legend and main text.

Thank you, it is corrected.

6) Fig. 3K. Mutant images are of higher magnification than WT images. For proper comparison, lower magnification mutant images should be shown here. There is space to actually show both, a low-magnification image of the entire area, and higher magnification pictures for the habenula for both WT and mutant at 2 different anterio-posterior levels.

Mutant and WT images are of the same magnification. It seems not to be, because in the mutant, the fibers of stria medullaris are not compact and extend on the thalamus, giving an impression that this is still the habenula. We followed the advice of the reviewer and included more images of different magnification in the Figure 3.

© 2020. Published by The Company of Biologists under the terms of the Creative Commons Attribution License (https://creativecommons.org/licenses/by/4.0/).

7) Main text about TCF7L2 direct binding: “suggesting that TCF7L2 acts directly as an activator rather than repressor”. TCF7L2 direct binding to upregulated genes also suggests direct role in repression. Without further evidence this suggestion should be revised.

In the revised version this part of the text is removed.
Reviewer 3 Comments for the authorand our answers:

1. The authors interpret their findings as to indicate that Tcf7l2 is a terminal selector gene for all thalamic neurons. This conclusion impinges mostly on analysis of prospective thalamo-cortical neurons and certainly the defects in electrical properties in glutamatergic neurons of the ventrobasal complex at P21 support this interpretation. However, Tcf7l2 is also expressed in the derivatives of the rTh domain of the second prosomere and these are fundamentally different cells in terms of neurotransmitter/neuropeptide expression, cell membrane properties and long-range axonal connectivity. Interestingly, several of the genes upregulated upon Tcf7l2 deletion at E18.5 are expressed in the rTh derivatives (e.g. Nkx2.2, Lhx5, Reelin) possibly highlighting an altogether different role of Tcf7l2 in this subdomain of prosomere 2. The authors should discuss whether they consider Tcf7l2 as required to provide pan-thalamic neuronal features that are shared among excitatory and inhibitory neurons alike, referring to the relevant experiment evidence.

The conclusion cannot be generalised to the rostral thalamus whose markers were not downregulated in Tcf7l2-/- embryos. We added the following comments:

Page 13 (Discussion)
Tcf7l2 knockout did not inhibit the expression of rostral thalamic markers, Nkx2-2, Sox14 and Lhx5, indicating a different role of TCF7L2 in this particular subdomain of prosomere 2.

2) Again, on the subject of how generalisable the findings are, the authors should discuss, and if additional evidence is available include it in the manuscript, whether the prospective midline thalamus should be included in their generalisation that Tcf7l2 is a terminal selector for the entire thalamus. Based on Cck RNA in situ data available on the Allen Brain Atlas, the Cck driver may not be active in certain thalamo-cortical nuclei, especially along the midline (i.e. PVN, reuniens, centromedial).

Tcf7l2 was partly knocked out also in the midline nuclei and AD. In the PVT and PF, majority of cells were TCF7L2-positive in the mutant mice. We show it now in more details in a supplemental figure S3. Nevertheless, the ChIP-seq data provided some evidence that the role of TCF7L2 in terminal selection can be generalised to the whole thalamus. We added the following comments:

Page 5 (Results)
In the resulting CckCre:Tcf7l2fl/fl mice, TCF7L2 was absent in most thalamic nuclei in adults, except for the PV and PF (Fig. S3). In the AD and midline nuclei, Tcf7l2 was partially knocked out.

Page 11 (Results)
The ChIP-seq peaks were detected (…) These genes were either broadly expressed in the thalamus (such as Cacna1g, Gabrd, Kcnc2, Syt7, Gabra4, Grm1, Grid2ip or Synpo2) or restricted to thalamic subregions (such as Grm1 to the anterodorsal and mediodorsal nuclei, Cacng3 to the PV and midline nuclei, Kcnab2 to the PF, AD and ventral nuclei, and Kcnd2 to the AD, PV and habenula). This confirmed that TCF7L2 is directly involved in the activation of genes that define pan-thalamic terminal identity and subregional identities in the thalamus.

Page 15 (Discussion)
The thalamus is molecularly distinguishable from other brain structures, but many thalamus-enriched genes are differentially expressed between thalamic nuclei or groups of nuclei (Nagalski et al., 2016; Phillips et al., 2019). TCF7L2 was proved to regulate genes that are broadly expressed in the thalamus and those that are specifically expressed in groups of thalamic nuclei. Although Tcf7l2 was not knocked out in PV and PF, and was less efficiently knocked out in the AD or midline nuclei, ChIP-seq analysis identified TCF7L2 peaks in excitability/synaptic genes whose expression is enriched specifically in these regions, implicating TCF7L2 in the direct control of subregional as well as pan-regional terminal selection in the thalamus.

© 2020. Published by The Company of Biologists under the terms of the Creative Commons Attribution License (https://creativecommons.org/licenses/by/4.0/).

3) Expression levels of Tcf7l2 are clearly different in certain areas: the authors acknowledge this for the rTh derivatives IGL and vLGN, but similar considerations should also apply to prospective thalamo-cortical nuclei, as can be seen for instance in Figure 1B WT panel, Figure 2B WT panel and Figure 2G WT P14 panel. The authors should articulate their “results” and “discussion” sections of the paper in view of potential differential requirements for Tcf7l2 in different thalamo-cortical nuclei.

This is a very interesting aspect. Indeed, TCF7L2 levels are lower in some parts of the thalamus in embryos. In adult, the level of TCF7L2 is similar between different thalamic nuclei (Nagalski et al., 2013). We commented it as follow:

Page 5 (Results)
At late gestation (...) Relatively lower levels of TCF7L2 were present in the ventrobasal complex (VB), nucleus reuniens, and recently identified perihabenula (Fernandez et al., 2018).

Page 13 (Discussion)
The mechanism of differential regulation of genes by TCF7L2 in subregions of prosomere 2 is not known. A differentiating factor might be the level of TCF7L2, which varies between thalamic nuclei during embryogenesis.

4) Images of thalamic tissue in the coronal plane are meant to illustrate an anterior and a posterior level. However, examples are fairly anterior and, in some cases, only one anterior section is provided. Figures 2G, Figure 6A (P60) and 6C, 6E should be revised to include examples of more caudal levels (i.e. at level of medial geniculate, posterior and full extension of the lateral geniculate complex).

We added a supplemental Figure S3 (to Fig. 2) with anti-TCF7L2 staining on a CckCre:Tcf7l2fl/fl brain; and Figure S5 (to Fig. 6) with sagittal sections of Vglut2 and Gad67 ISH, and series of coronal sections for Rora and Cav3.1.

5) The authors should comment on the relationship between the prethalamic cells visible at E12.5 in Figure 1A and the vLGN. Can the authors confidently say that all the Tcf7l2 positive cells in the vLGN are prosomere 2 derived?

No, we cannot say this, but we can speculate. It is possible that these are migrating cells of the rostral thalamus that finally contribute to the IGL/vLGN. To include this speculation, we edited the description of these results as follow:

Page 5 (Results)
We also observed several TCF7L2-positive cells in the prethalamus. Possibly, these cells migrate from the rostral thalamus to take part in the formation the intergeniculate leaflet and ventral lateral geniculate nuclei that derive from prethalamic and rostral thalamic progenitors (Jeong et al., 2011).

6) Can the authors comment on the Tcf7l2 negative area lateral to the habenular complex visible clearly in Figure 1B and its inset n.1? The area corresponds to the recently described perihabenula (Fernandez et al. Cell 2018)- a novel nucleus of the thalamus. Could this nucleus depend on a distinctive differentiation program?

This area is rather TCF7L-low than TCF7L2-negative. We believe that this question is addressed in our answer to the point 3.

7) Optional - Would the authors be in the position to add data to Figure 2 illustrating Cck-cre activity, using a suitable reporter line? This is not essential but would help refining the overall interpretation of the gene expression data.

We added fluorescent images of brain slices from CckCretdTomatofl/fl reporter mice as a supplemental
Figure S2 and the following description:

© 2020. Published by The Company of Biologists under the terms of the Creative Commons Attribution License (https://creativecommons.org/licenses/by/4.0/).

Page 5 (Results)
The expression from the CckCre locus, visualised in CckCre:tdTomatofl/+ reporter line, was high in lateral parts of the thalamus, and lower in thalamic medial and midline parts, including anterodorsal (AD), paraventricular (PV) and parafasicular (PF) nuclei (Fig. S2).

8) Figure 3C. There appears to be an increase in the number of Pou4F1 cells, not just a spread into thalamic territory. The authors should comment on this finding and also address whether they think there is a conversion of thalamic neurons into epithalamic ones or an intermingling of thalamic and epithalamic neurons.

The number of POU4F1-positive cells is visibly higher on E12.5 in Tcf7l2-/- embryos, meaning that either more POU4F1-positive cells are generated or they are generated earlier than in wild type condition. It is difficult to say if the number of these cells is higher at late gestation, because they are very densely packed in wild type habenula and scattered in the thalamo-habenular area in the mutant mice. This sparse distribution of POU4F1-positive cells indicates unusual migration rather than identity switch. We discussed it in more detail as follow:

Page 6 (Results)
The number of POU4F1-positive cells visibly increased, suggesting that prosomere 2 cells more readily adopted habenular fate in Tcf7l2-/- embryos at this stage.

Page 7 (Results)
Sparse distribution of these cells, in particular POU4F1-positive cells, pointed to their unusual migration rather than identity switch.

Page 13 (Discussion)
An apparent increase in the number of POU4F1 cells in Tcf7l2-/- embryos on E12.5 suggests that TCF7L2 could play a regulatory role in cross-repressing thalamic and habenular identities, by promoting thalamic fate, in agreement with a previous conclusion (Lee et al., 2017). However, decreased expression of sub-habenular markers, e.g., Etv1 and Nr4a2, as well as a thalamo-habenular marker Lef1, and sub-thalamic markers, e.g., Foxp2, Prox1 and Rora in Tcf7l2-/- embryos on E18.5 indicates that TC7L2 plays a positive role in the development and diversification of both thalamic and habenular identities.

Page 14 (Discussion)
However, a comparison between the effects of Tcf7l2 and Pou4f1 knockouts in the habenula is not straightforward. Anatomical impairments were much more severe in Tcf7l2-/- embryos, but much of this phenotype may be attributed to secondary effects that result from the spread of POU4F1-positive cells throughout lateral part of prosomere 2 in Tcf7l2-/- embryos.

9) Figure 3F (but also 4B) seems to suggest that the obvious migratory defect in the prethalamic Pax6 population (lateral thalamus/prethalamus) is cell-extrinsic. Can the authors comment further on that?

We added a following comment:

Page 14 (Discussion)
Considering that Pax6-positive prethalamic cells do not express Tcf7l2 in wild type embryos, abnormal intermingling of these cells into thalamic territory must be cell non-autonomous. The same may apply to the impaired segregation of rostral thalamic and habenular cells. Mechanisms that regulate cell migration and nucleogenesis in the diencephalon are poorly understood. We speculate that misexpression of cell adhesion genes in the thalamus, such as ectopic expression of Reln and decreased expression of thalamus-specific genes Cdh6, Cdh8, and Cntn6, could turn the thalamus into a permissive environment for cells migrating from the neighboring Reln-positive structures, i.e., prethalamus, rostral thalamus, habenula and, possibly, pretectum.

10) Figure 3J,K: similar data already published by Lee et al. Dev Bio 2017. I am not convinced that panels J and K should be included.

© 2020. Published by The Company of Biologists under the terms of the Creative Commons Attribution License (https://creativecommons.org/licenses/by/4.0/).

We would prefer to keep these results because they illustrate anatomical impairments that are underlined by altered expression of cell adhesion and axon guiding genes in Tcf7l2 KO embryos. These results were published by Lee, but we noticed also new things. We show that the fasciculus retroflexus and stria medullaris were severely disorganised, whereas it was previously reported that the fasciculus retroflexus was missing and stria medullaris were normal (on E16.5). However, we changed the order of these paragraphs. Now the description of boundaries is not interrupted and we believe it reads better.

11) Figure 5. The authors should measure enrichment for rTh and cTH specific genes in the downregulated and upregulated cluster. Based on a scan-through the gene list, it would appear that while epithalamic and cTh genes are downregulated in the Tcf7l2 knockout, rTh are upregulated. If so, how can this be interpreted?

Yes, the RNA-seq analysis showed higher levels of rTh-specific transcriptsin Tcf7l2-/- embryos. However, this might result from lateral elongation of the rTh into the causal thalamic area, and consequently a higher proportion of rTh mRNA in RNA-seq samples. While the ectopic expression of reelin transcripts in the thalamus is genuine, ISH staining does not show stronger or ectopic signal from the rTh markers Nkx2-2 and Sox14.

Page 7 (Results)
The rostral thalamus area was elongated laterally (Fig. 4C).

Page 8 (Results)
An increased level of the rostral thalamus markers Nkx2-2, Sox14 and Lhx5 was also observed, and was likely caused by the expansion of this domain into the caudal thalamic area

Page 13 (Discussion)
Tcf7l2 knockout did not inhibit the expression of rostral thalamic markers, Nkx2-2, Sox14 and Lhx5, indicating a different role of TCF7L2 in this particular subdomain of prosomere 2.

12) Figure 7B. The Venn diagram should be modified to include all ChIP enriched genes, not just those that are contained within the transcriptionally regulated groups.

The Venn diagrams have been adjusted to include all ChIP-Seq genes and all RNA-seq data.

13) Discuss putative Tcf7l2 transcriptional targets identified here in relation to transcriptional targets previously identified in Lee et al. Dev Bio 2017 and LEF1/TCF targets previously identified by the senior author, particularly Cacna1G Wisniewska et al. J Neurosci 2010.

Lee et al. analysed TCF7L2 targets at different time point of development (E12.5 and E14.5). However, we agree that it would be an interesting addition. We discussed it as follow:

Page 14 (Discussion)
A previous research showed that the aberrant growth of thalamocortical axons toward the hypothalamus instead of the ventral telencephalon in Tcf7l2-/- embryos resulted from unresponsiveness of thalamic cells to Slit repulsive ligands, due to decreased expression of genes that encode Slit receptors Robo1 and Robo2 (Lee et al., 2017). We did not observe any changes in the levels of Robo1 and Robo2 mRNA. The expression of these genes is specific for prosomere 2 only at earlier stages; hence it may not depend on TCF7L2 at late gestation. Instead, we observed decreased expression of genes that encode habenular axon-navigating molecules Robo3 and Rgma and thalamic axon-navigating molecules that are later induced and subregion-specific, e.g., Ntng1, EPHA1, 3, 4, 8. Eph receptor A4 (EPHA4) regulates topographical sorting of VB axons in the ventral telencephalon at late gestation (Dufour et al., 2003). This implicates TCF7L2 in controlling the sequential steps of thalamocortical axon navigation and subregional sorting.

We also commented on our previous research as follow:

Page 15 (Discussion)
This is consistent with our previous in silico predictions and ChIP-qPCR which showed that β-catenin, which is a cofactor of LEF1/TCF transcription factors, interacts with promoters of several

© 2020. Published by The Company of Biologists under the terms of the Creative Commons Attribution License (https://creativecommons.org/licenses/by/4.0/).

excitability/synaptic genes, including in particular Cacna1g (Wisniewska et al., 2010; Wisniewska et al., 2012).

Methodological considerations (mandatory revision in my view):
14) There is insufficient data on the quality of the material used for the ChIP experiment. How specific is the TCf7l2 antibody used? The authors should show biochemical validation of the IP and include a negative control, for instance the TCf7l2 knockout thalamus sample, where the IP shouldn't pull down any DNA.

We are aware of methodological challenges associated with ChIP-Seq. The specificity of an antibody is crucial. We did not stress this enough in the paper. For the ChIP-seq we used an anti-TCF7L2 antibody (C48H11; Cat. no. 2569, Cell Signaling) that has been previously validated for ChIP-seq experiments in other studies and by the ENCODE consortium. We also validated the specific lot of the antibody.
Validation of the antibody according to the ENCODE recommendation, from 2016, can be performed in various ways:

https://www.encodeproject.org/documents/c7cb0632-7e5f-455e-9119-46a54f160711/@@download/attachment/ENCODE_Approved_May_2016_TF_Antibody%20Characterization_Guidelines.pdf
It is recommended to perform one primary and one secondary validation. The best primary validation method is Western blot. This analysis is included in Fig. 2C and D. Here we show an additional Western blot on nuclear lysate with two bands representing two isoforms of TCF7L2. Secondary validation methods recommended by ENCODE include: 1. Western blot on knockout samples; 2.Comparison with ChIP-Seq performed with a different antibody; 3. Immunoprecipitation with an epitope-tagged version of the protein; 4. Motif enrichment analysis. The Western blot shown on the Fig. 2C and D represents both wild type and Tcf7l2 KO samples, showing the appropriate specificity of the antibody.
W explained it as follow:

Page 10 (Results)
We used the same antibody that we used for Western blot and immunofluorescence/immunohistochemistry in this study, and which was validated with samples from the mutant animals (Fig. 2B-G). This antibody was previously used by other authors on different cell types (Frietze et al., 2012; Geoghegan et al., 2019; Norton et al., 2011).

In addition, as a negative control, we sequenced DNA precipitated with normal rabbit IgG. This analysis produced low number of peaks with only 4 peaks overlapping with anti-TCF7L2 peaks (these peaks were subtracted).

Now, we added two other validations:
1. Because the TCF7L2 binding motif is well characterised, in the revised version of our study we additionally performed motif enrichment analysis on the ChIP-seq peaks with the AME algorithm from the MEME suit. This analysis, included in Fig. 7A, was concluded as follow:

Page 11 (Results)
Analysis of motif enrichment (the AME algorithm from the MEME suite) showed significant overrepresentation of the consensus motif for TCF7L2 in the sequences bound by the anti-TCF7L2 antibody. This motif was detected in almost 85% of the ChIP-seq peaks (Fig. 7A), validating our experiment.

2. Reviewer 3 suggested a ChIP-seq on samples from the knockout animals as a validation method. Although ENCODE has withdrawn this method from their recommendations, we were also convinced that this is the best control. We have had already collected samples from the thalami isolated from CckCre:Tcf7l2fl/fl mice. Luckily, because this material was already waiting for sequencing, we could complete this analysis despite the lockdown rules. The result of this validation is described as follows:

Page 11 (Results)

© 2020. Published by The Company of Biologists under the terms of the Creative Commons Attribution License (https://creativecommons.org/licenses/by/4.0/).

In addition, we used a thalamic sample from CckCre:Tcf7l2fl/fl mice. 94,3% of the peaks identified in the wild type condition were not detected in this sample, proving specific target recognition in our assay.

15) Figure 5 A. there are inconsistencies with the first sample out of the three knockout replicates. Does this reflect biological variation or the dissection method? The description of the dissection method at E18.5 and P60 should be provided in greater detail. Where there any post-dissection controls carried out to assess

The dissection method is now described and illustrated in a supplemental Figure S7 - see our response to Reviewer 1 point 3. We had no post-dissection verification. The whole procedure was done by one person who was already experienced in brain sectioning, in particular the thalamic region. These sections are contaminated with surrounding structures (prethalamus and pretectum) to some extent, this is why we decided to use a fold change cut-off of log2 <-0.4 and >0.4 (in addition to statistical cut-off), to decrease the number of potentially false positive results. Moreover, for E18.5 samples, RNA seq was run twice with independent sets of 3 biological replicates, giving basically the same result. Regarding the “outlier sample”, we think that it reflects a real variation, because knockout thalami showed different penetrance of the phenotype, visible even on macroscopic level.

Please, note that we did several additional corrections:

1. We corrected the number of the differentially expressed genes on E18.5. The previous number corresponded to the log2 fold change cut-off at 0.5, whereas in the paper we set the cut-off at 0.4. For the same reason we corrected a plot with differentially expressed transcription factor genes on P60.
2. We observed that TCF7L2 binding sites were overrepresented in introns and decided to adjust the annotation algorithm so that it would favor the annotation to genes within which the peaks are localised instead of the annotation to putative regulatory elements. With this adjustment, 45% of downregulated and 31% of upregulated genes identified in RNA-seq were also discovered in ChIP-seq data. In the previous version, it was 38% and 22%, respectively.
We explain it as follow:

Page 22 (Materials and Methods)
Because TCF7L2 was enriched in intronic regions, we adjusted the annotation algorithm to prioritise the association of peaks with introns.

Page 11 (Results)
The peaks annotated to genes that were expressed in the thalamus on P60 were most frequently localised in intronic regions that may act as intragenic enhancers (Fig. S6B and Table S5). The remaining peaks (i.e., annotated to non-expressed genes) were mainly annotated to predicted genes and pseudogenes, and were located in distal intergenic regions, suggesting that they represent distal enhancers of unidentified genes.

3. We also included a discussion with a recent paper:

Page 16 (Discussion)
A recent research reported that cells in a thin superficial portion of the thalamus switched to GABAergic identity in Tcf7l2-/- embryos, showed by the colocalisation of Gad1 and Gbx2-driven TdTomato signal (Tran et al., 2020). However, the staining resolution does not allow concluding that the signals colocalised in the same cells; and according to the most recent research the origin of thalamic GABAergic cells may be assigned to prethalamic, rostral thalamic and even pretectal domains Jager et al., 2016; Puelles et al., 2020). More importantly, normal pattern of Vglut2 and Gad1 expression in mice with the postnatally induced knockout of Tcf7l2 demonstrates that TCF7L2 does not play a role in maintaining glutamatergic identity in thalamic neurons.

© 2020. Published by The Company of Biologists under the terms of the Creative Commons Attribution License (https://creativecommons.org/licenses/by/4.0/).

Second decision letter

MS ID#: DEVELOP/2020/190181

MS TITLE: TCF7L2 regulates postmitotic differentiation programs and excitability patterns in the thalamus

AUTHORS: Marcin Andrzej Lipiec, Joanna Bem, Kamil Koziński, Chaitali Chakraborty, Joanna Urban-Ciecko, Tomasz Zajkowski, Michal Dabrowski, Lukasz Mateusz Szewczyk, Angel Toval, Jose Luis Ferran, Andrzej Nagalski, and Marta Barbara Wisniewska
ARTICLE TYPE: Research Article

I am delighted to tell you that your manuscript has been accepted for publication in Development, pending our standard ethics checks.

© 2020. Published by The Company of Biologists under the terms of the Creative Commons Attribution License (https://creativecommons.org/licenses/by/4.0/).
